# Mapping of flumioxazin tolerance in a snap bean diversity panel leads to the discovery of a master genomic region controlling multiple stress resistance genes

**DOI:** 10.3389/fpls.2024.1404889

**Published:** 2024-07-02

**Authors:** Ana I. Saballos, Matthew D. Brooks, Patrick J. Tranel, Martin M. Williams

**Affiliations:** ^1^ Global Change and Photosynthesis Research Unit, United States Department of Agriculture–Agricultural Research Service, Urbana, IL, United States; ^2^ Department of Crop Sciences, University of Illinois, Urbana, IL, United States

**Keywords:** *Phaseolus vulgaris*, herbicide tolerance, abiotic stress resistance, xenobiotic detoxification, flumioxazin, PPO-inhibiting herbicide, plant breeding

## Abstract

**Introduction:**

Effective weed management tools are crucial for maintaining the profitable production of snap bean (*Phaseolus vulgaris* L.). Preemergence herbicides help the crop to gain a size advantage over the weeds, but the few preemergence herbicides registered in snap bean have poor waterhemp (Amaranthus tuberculatus) control, a major pest in snap bean production. Waterhemp and other difficult-to-control weeds can be managed by flumioxazin, an herbicide that inhibits protoporphyrinogen oxidase (PPO). However, there is limited knowledge about crop tolerance to this herbicide. We aimed to quantify the degree of snap bean tolerance to flumioxazin and explore the underlying mechanisms.

**Methods:**

We investigated the genetic basis of herbicide tolerance using genome-wide association mapping approach utilizing field-collected data from a snap bean diversity panel, combined with gene expression data of cultivars with contrasting response. The response to a preemergence application of flumioxazin was measured by assessing plant population density and shoot biomass variables.

**Results:**

Snap bean tolerance to flumioxazin is associated with a single genomic location in chromosome 02. Tolerance is influenced by several factors, including those that are indirectly affected by seed size/weight and those that directly impact the herbicide's metabolism and protect the cell from reactive oxygen species-induced damage. Transcriptional profiling and co-expression network analysis identified biological pathways likely involved in flumioxazin tolerance, including oxidoreductase processes and programmed cell death. Transcriptional regulation of genes involved in those processes is possibly orchestrated by a transcription factor located in the region identified in the GWAS analysis. Several entries belonging to the Romano class, including Bush Romano 350, Roma II, and Romano Purpiat presented high levels of tolerance in this study. The alleles identified in the diversity panel that condition snap bean tolerance to flumioxazin shed light on a novel mechanism of herbicide tolerance and can be used in crop improvement.

## 1 Introduction

Snap bean (*Phaseolus vulgaris* [L.]) producers face a significant challenge due to weed contamination in mechanically harvested beans. The presence of waterhemp (*Amaranthus tuberculatus*) exacerbates the issue as the fragile stem pieces are hard to differentiate from the snap bean pods, leading to contamination. Waterhemp is among the most common and troublesome weeds in North American crop production systems (Van Wychen, 2015) due to its high fecundity, discontinuous emergence pattern, rapid growth rate, and resistance to herbicides of multiple modes-of-action groups. Unlike major crops such as soybean (*Glycine max* (L.) Merr.), transgenic herbicide resistance is not available for snap bean production, and chemistries suitable for use on snap bean crop are limited. Expanding the availability and use of herbicides from different modes of action is one approach to improve weed control and delay herbicide resistance in weed populations (Gage et al., 2019).

Flumioxazin effectively controls waterhemp and multiple other broadleaf weeds (Niekamp et al., 1999); however, it is not labeled for use in snap bean. One hurdle to registering an herbicide on a new crop is the unknown level of crop tolerance to the candidate herbicide. The extent of naturally occurring tolerance to flumioxazin in snap bean cultivars and its genetic basis are unknown.

Flumioxazin belongs to the group of protoporphyrinogen oxidase (PPO)-inhibiting herbicides [Flumioxazin, National Center for Biotechnology Information, (2024)]. Protoporphyrinogen IX oxidase (EC 1.3.3.4) is an oxygen-dependent enzyme essential for the biosynthesis of chlorophyll, catalyzing the oxidation of protoporphyrinogen IX to protoporphyrin IX in the chloroplast (Poulson and Polglase, 1975). When PPO is inhibited, its substrate is exported to the cytoplasm, oxidizing it into protoporphyrin IX. In the presence of light, protoporphyrin IX produces reactive oxygen species (ROS), resulting in the loss of chlorophyll and carotenoids, degradation of lipids and proteins, and disruption of cell membranes (Nagano, 1999; Maurya, 2020). As a soil-applied herbicide, flumioxazin is in contact with the seedling from the start of germination; therefore, defense mechanisms must be active in germinating seeds. Plants exhibit herbicide tolerance through target-site and non-target-site resistance mechanisms (TSR and NTSR, respectively). While TSR results from genetic mutations in the herbicide targets, or from increased target gene copies or its expression (Gaines et al., 2020), NTSR occurs via various physiological and biochemical mechanisms. Target-site resistance to PPO inhibitors due to mutations in one of the two isoforms of PPO (Lermontova et al., 1997) has been reported in multiple Amaranthus species including *A. artemisiifolia*, *A. tuberculatus*, *A. palmeri*, *A. retroflexus*, and other weed species including *Eleusine indica*, and *Euphorbia heterophylla* (Patzoldt et al., 2006; Lee et al., 2008; Rousonelos et al., 2012; Giacomini et al., 2017; Bi et al., 2020; Mendes et al., 2020; Du et al., 2021).

Non-target-site herbicide resistance alters physiological processes, including absorption, translocation, sequestration, and metabolism. These processes provide defense against a wide range of xenobiotic compounds. Metabolic tolerance is likely to be the mechanism of tolerance to sulfentrazone, another PPO-inhibiting herbicide, in snap bean (Saballos et al., 2022). It can be mediated by detoxification of the molecule and by ameliorating its effects in the cell (Edwards et al., 2011; Cavé-Radet et al., 2020). Mechanisms of NTSR are more complex than TSR and can impart cross-resistance to herbicides with different modes of action (Jugulam and Shyam, 2019). Flumioxazin-induced ROS production results in oxidative stress. The oxidative stress phenomenon accompanies nearly all plant stresses (Demidchik, 2015) and plants have developed mechanisms to sense and ameliorate it. Mechanisms linking processes in respiration, photosynthesis, plant hormones, antioxidant enzymes, antioxidant compounds and chaperone proteins protect the cells against oxidation (Lee et al., 2000; Yamauchi et al., 2012; Luhua et al., 2013; Sah et al., 2016; Mahmood and Dunwell, 2020; Maurya, 2020; Dumanović et al., 2021). Those mechanisms are ultimately controlled at the transcriptional level, with transcription factors (TF) coordinating downstream gene expression. Understanding the genetic basis and mechanisms of NTSR is crucial for managing weed herbicide resistance evolution (Délye, 2012) and aiding in breeding herbicide-tolerant crops.

Genome-wide association study (GWAS) is used to study traits without a known genetic structure. This approach involves scanning the genome of a species to identify markers with statistical associations with traits of interest (Manolio, 2010). GWAS is able to identify genomic regions containing loci of moderate to large effect (Lipka et al., 2015; Delfini et al., 2021). This approach has been successful identifying genomic regions associated with NTSR in crop and weed species. Sehgal et al. (2024) reported four SNPs significantly associated with reduced sensitivity to an acetyl CoA carboxylase -inhibiting herbicide in a diverse population of *Digitaria insularis* from Brazil. Candidate genes located in the associated regions were postulated to have functions in herbicide detoxification and in vacuolar sequestration-based degradation pathways. Using a snap bean diversity panel, we identified multiple genomic regions associated with variation in sensitivity to the PPO- inhibiting herbicide, sulfentrazone (Saballos et al., 2022). Genes with possible functions in NTSR, including those encoding cytochrome P450 enzymes and ABC transporters, were located in the associated intervals.

GWAS-identified regions may contain multiple genes depending on the population used and marker density. Additional approaches often are needed to characterize the possible roles of candidate genes in the expression of the phenotype. Transcriptional profiling and co-expression network analysis can be helpful in the identification of gene modules and key genes responsible for a particular condition. The predicted functions of the genes composing an associate module shed light on the biological processes involved in the expression of the phenotype. Weighted Gene Correlation Network Analysis (WGCNA) is a technique widely used for finding groups of genes, called modules, that have highly correlated expression levels across samples (Langfelder and Horvath, 2008). Through this approach, co-expression networks are constructed. Gene modules associated with various traits have been successfully detected in Arabidopsis (*Arabidopsis thaliana*), rice (Oryza sativa), maize (*Zea mays*), soybean, tomato (*Solanum lycopersicum*), sugarcane (*Saccharum officinarum*), and aspen (*Populus* sp.) (DiLeo et al., 2011; Shaik and Ramakrishna, 2013; Gerttula et al., 2015; Choe et al., 2016; Ferreira et al., 2016; Das et al., 2017).

This study aimed to explore the genetic basis of snap beans’ tolerance to flumioxazin. We sought to identify the specific genomic regions linked to this tolerance and gain a better understanding of the biological processes that contribute to it. To achieve these objectives, we used a diversity panel to evaluate the levels of flumioxazin tolerance within the crop, combined with genome-wide association mapping and gene expression data to reveal the genetic control of this trait.

## 2 Materials and methods

### 2.1 Phenotype evaluation, summary statistics and genome-wide association studies

#### 2.1.1 Germplasm

377 genotypes of the Snap bean Association Panel (SnAP) were used in this study (Hart et al., 2015). SnAP represents the diversity of snap beans grown in the US over the last century. The original SnAP population was genotyped using Genotyping by Sequencing (GBS) and aligned to the reference Andean G19833 *P. vulgaris* v2.1 genome sequence (Schmutz et al., 2014). A total of 20,619 SNPs with a minimum allele frequency of 5% were included in the analysis (Soler-Garzón et al., 2023).

#### 2.1.2 Field experiment

The study was conducted at the University of Illinois Vegetable Crop Farm near Urbana, IL. Experiments were planted on July 8^th^, 2021 and June 2^nd^, 2022, for the first and second year of the experiment. The experimental design was a strip plot with three blocks (replications) as described in Saballos et al. (2022). Plots received one of two levels within 24 hours after planting: flumioxazin at 378 g a.i. ha^-1^ or a nontreated control. The flumioxazin rate, twice the recommended use rate in soybean for soil at the location, was chosen to represent an overlap of the highest possible practical rate for maximum weed control.

Genotype treatment plots consisted of single rows (76-cm spacing) of individual genotypes transecting both herbicide treatment strips. Each genotype by herbicide treatment subplot was 2.4 m in length planted with 30 seeds to a depth of 2.5 cm.

Prior to planting, seed weight was taken from a random sample of 100 seeds per genotype. The 100-seed weight of the seed lots of both years were averaged to represent the seed weight of each genotype. Seed weight was used as a proxy measurement of seed size (e.g., Giles, 1990; Roy et al., 1996).

#### 2.1.3 Field data collection

For each plot, emerged seedlings with green tissue at the meristem were counted to determine plant density (PD). This measure reflects a combination of germination and seedling establishment. At the same time, individual plant shoot biomass was determined. The shoot tissue from three randomly selected plants from each subplot was harvested. Shoots were dried until constant weight to determine biomass plant^-1^ (BP). This measure gives an estimate of seedling growth. The level of tolerance of the genotypes was calculated from the above measures by expressing the values of the traits in the treated plots as percentage of the values in the control plots of the same genotype within the same block (named PDperc and BPperc). The PD percentage of the control plots (PDcontrol) was calculated as the number of seedlings emerged in the control plots divided by the number of planted seeds (30), expressed as percentage. BPcontrol was the dry shoot biomass per plant of the genotypes in the control plots.

Daily rainfall and temperature were obtained from a weather station located within 1 km of the experiments (Illinois State Water Survey, Champaign, IL). Growing degree days (GDD) were calculated using the formula:


$$\left[\frac{T\text{max}-T\text{min}}{2}\right]-Tbase$$

Where Tmax is the daily maximum air temperature, Tmin is the daily minimum air temperature, and Tbase is the minimum development threshold that must be exceeded for growth to occur. The Tbase was set a 10˚C for common bean (Dethier and Vittum, 1963).

#### 2.1.4 Field data analysis

PDperc and BPperc were analyzed by ANOVA with the aov() function in R studio using the model:


$$y_{ilk}=\;\mu \;+\;G_{i\;}\;+\;Y_{l}+\;{\left(GY\right)}_{il}+\;B{\left(Y\right)}_{k\left(l\right)}\;+\;\epsilon_{ilk}$$

where *Y_ilk_* is the trait value of the plot in the *k^th^* block in the *l^th^* year, with the *i^th^* genotype, *µ* is the overall mean of the experiment, *G_i_* is the main effect of the *i^th^* genotype, *Y_l_* is the main effect of the *l^th^* year, *(GY)_il_* is the interaction effect between the *i^th^* genotype and the *l^th^* year, *B(Y)_k(l)_* is the effect of the *k^th^* block nested within the *l^th^* year and *ϵ_ilk_* in the error term associated with plot in the *k^th^* block in the *l^th^* year with the *i^th^* genotype. All effects were declared significant at α=0.05.

Broad-sense heritability for PDperc and BPperc was calculated as a function of variance components, as described in Holland et al., 2002. Variance components were obtained by fitting a linear model with the lmer() function using the model above. Marker-based estimates of narrow-sense heritability were obtained using the method proposed by Kruijer et al. (2015), using a mixed model in which genetic relatedness is estimated from genetic markers, and the trait value at the individual plot level. The model was implemented with the R package Heritability (Kruijer et al., 2023).

Pearson correlation coefficients expressing the linear relationship between the best linear unbiased predictions (BLUPs) of the traits were calculated using the procedures cor and Rcorr of package Hmisc (R studio). Data were visualized using the package Corrplot.

#### 2.1.5 Data preparation and GWAS analysis

Normality of the raw data was assessed using the R rstatix package, Shapiro_test(). Box-Cox transformation was applied when necessary to improve the normality of the distribution of the trait values (Box and Cox, 1964). The optimal Lambda values for each trait were calculated using the function boxcox() of the MASS package (R studio). The optimal value is the one which results in the best approximation of a normal distribution curve for transformed trait data. For traits with negative effect values, a constant was added to the data to allow for the calculation of Lambda, and the transformation was applied to the raw values.

BLUPs were determined for the tolerance traits from the transformed datasets using the function lmer() of the lme4 package, with genotype and block as random effects in the model for the individual year analysis, and genotype, year, genotype by year interaction and block within year as random effects for the joint analysis. The conditional means of the genotypes were extracted using the function ranef () of the lm4 package.

The best linear unbiased predictions were used as input for the GWAS model. Forward model selection using the Bayesian information criterion (BIC) was used to determine the optimal number of principal components (PCAs) to include in the GWAS models for each trait.

As a correlation between seed weight and tolerance traits was observed in the field experiments, GWAS analysis was conducted with seed weight as covariate to detect tolerance-associated loci independent of the effect of seed weight.

The statistical model used in the GWAS was the multi-locus mixed model (MLMM) as implemented in GAPIT v.3 (Wang and Zhang, 2021). In the MLMM (Segura et al., 2012), associated markers are fitted as cofactors for marker test. The cofactors are adjusted through forward inclusion and backward elimination in the regression model. The Benjamini and Hochberg (1995) procedure was used to control multiple testing. The kinship matrix used for analysis was calculated in GAPIT using the VanRaden method (VanRaden, 2008). The models included the optimal number of PC calculated by BIC, the kinship matrix, and seed weight as covariate. The analysis was run independently for each year, and jointly for both years.

Linkage disequilibrium decay was calculated using the pairwise *r^2^* of SNPs within each chromosome. Using the software package TASSEL Version 5.0 (Bradbury et al., 2007), the genotypic map was thinned to a minimum distance of 2 KB between SNPs using the option “Thin Sites by Position” of the “Data” tab. The *r^2^* between the SNPs was calculated with the option “Linkage disequilibrium” within the “Diversity” group of the “Analysis” tab. The resulting matrix was exported to R studio for manipulation and plotting. The distance between SNPs was divided in bins of 10 kbp, and the average *r^2^* of the bin was calculated. A line plot was created using the ggplot2 package with the average *r^2^* of the bin in the x-axis, and the distance between the SNP at the start of the bin in the y-axis.

#### 2.1.6 Genomic prediction

The two-year averages of the PDperc and BPperc were used to estimate the breeding value of the snap cultivars using the gBLUP model (Zhang et al., 2007) as implemented in GAPIT. The average seed weight of the lots was used as a covariate in the analysis. The Pearson correlation of the predicted phenotype and the observed phenotype was calculated to determine the predictive ability (PA). To evaluate the accuracy of the prediction, a genomic prediction model was generated using the kinship matrix developed from all the markers and a random set of 80% of the phenotypes (reference set). The genotypes were not included in the model generation constituted the inference set. The model was used to predict the phenotype of the genotypes in the reference and inference set. The correlation between the original phenotypes and predicted phenotypes in the reference and inference set was recorded. The procedure was repeated 1000 times with randomly selected reference and inference sets to calculate the average accuracy.

#### 2.1.7 Effect of seed weight and significant SNP status on the tolerance phenotype

Scatter plots were used to visualize the relationship between seed weight and PDperc/BPperc values within groups of cultivars carrying the positive or negative effect allele of the significant SNPs. Box plots were used to visualize the effect of the allelic status at the significant SNP on the PDperc and BPperc values of the cultivars.

#### 2.1.8 Haploblock analysis

Linkage disequilibrium blocks surrounding the significant SNPs were determined using the option extractHaplotype (Barrett et al., 2004) in HAPPI-GWAS package (Slaten et al., 2020). extractHaplotype calculates pairwise LD between each significant SNP identified in GWAS and every neighboring SNP in a window. For the analysis, a window size of 10000 kbp was used. The haploblocks were defined as regions in which the upper and lower 95% confidence bounds on normalized measure of allelic association (D’) between pairs of SNPs are >0.98 and >0.70, respectively (Gabriel et al., 2002).

### 2.2 Differential expression, mutation analysis and GO term enrichment analyses

#### 2.2.1 Plant material

Twelve cultivars were included in the experiment. Tolerant response was represented by six cultivars with PDperc > 62% and BPperc > 50%. Six cultivars with PDperc< 16% and BPperc< 47% represented sensitive response. To control for the effect of seed weight, the selection was restricted to genotypes with 100-seed wt. between 42.44 and 50.33 g based on the average of the 2021 and 2022 seed lots. The genotype name, market class, seed weight, allelic status at associated SNP marker and response to flumioxazin of the genotypes included are presented in **Supplementary Table 1**.

Seeds were sown in 27.8 × 53.3 cm flats containing fine quartz sand. Thirty seeds of each cultivar were planted 1 cm deep. Flats were kept at 26°C and watered regularly to maintain soil moisture. Whole seedlings were harvested when the cotyledons had fully emerged. Seedlings were flash-frozen in liquid nitrogen immediately after harvest. Herbicide was not applied, with the assumption that differential gene expression contributing to tolerance would be constitutive (Giacomini et al., 2018).

#### 2.2.2 RNA extraction

Total RNA was obtained from four independent biological repeats, each pooled from 4 plants (16 plants total per genotype). Each frozen sample was ground to a fine power using a coffee grinder cooled with dry ice and extracted using E.Z.N.A Plant RNA kit I (Omega Bio-tek, Norcross, CA), and on-column DNase digestion was performed according to the manufacturer’s protocol. Paired reads were sequenced on a NovaSeq S4 flow cell (Illumina, San Diego, CA). Processing of the initial reads was performed using the Illumina analysis pipeline. Additional filtering was performed by removing adaptor sequences and low-quality bases.

#### 2.2.3 Differential expression analysis

After demultiplexing, adapter trimming, and filtering for low quality reads using the BBDuk tool in the BBtools suite of bioinformatic tools, version 38.94 (Bushnell, 2022), reads were aligned (Haas, 2003) to the *Phaseolus vulgaris* v2.1 reference genome (DOE-JGI and USDA-NIFA, 2023), downloaded from Phytozome v13 (Goodstein et al., 2012). Gene-level counts were obtained using featureCounts (Liao et al., 2014). Differentially expressed (DE) genes were identified between sensitive and tolerant lines using DESeq2 (Love et al., 2014). Differentially expressed genes between the tolerant and sensitive cultivars were identified with a cutoff of fold-change ≥ 1.5 and adjusted *p*-value ≤ 0.1 (Benjamini and Hochberg, 1995).

##### 2.2.3.1 GO term analysis of differentially expressed genes

An enrichment analysis was performed to discover significantly overrepresented functional categories. We functionally classified DEG according to GO terms using agriGO v.2 (Tian et al., 2017), using the Hochberg multi-test adjusted *p*-value of the F statistic< 0.01. Only significant GO term represented for at least 3 entries in the query list were considered. The GO annotation system is based on three structured vocabularies that describe gene products in terms of their associated biological processes, cellular components, and molecular functions. Upregulated and downregulated DEG were analyzed separately. The results were visualized using the online tool REVIGO (Supek et al., 2011).

#### 2.2.4 Variant discovery and annotation

Read manipulation, alignment and variant calling were performed with bioinformatic tools available at the University of Illinois Carl R. Woese Institute for Genomic Biology High Performance Computing cluster. Reads were filtered and trimmed for quality using the BBDuk tool (Bushnell, 2018). The 15 right-most bases were forced trimmed, and additional low-quality bases were trimmed until the overall read quality was above 30. Filtered reads were aligned using HISAT2 2.2.1 (Kim et al., 2019) to the *P. vulgaris* V2.1 reference genome. Only reads that mapped uniquely to one position in the genome were considered. The output BAM files were filtered to eliminate reads with mapping quality below 30 using SAMtools 1.12 (Li et al., 2009). BCFtools 1.12 (Danecek et al., 2021) was used to generate the genotype likelihood at each genomic position for the 48 samples and identify SNPs. For BCFtools mpileup command, -C 50 option was used to downgrade the mapping quality for reads containing excessive mismatches. For BCFtools call command, a p-value threshold of 0.0001 was used. The resulting VCF file was filtered using BCFtools filter command for calls of quality ≥ 40, supported for 8 or more reads. SNP markers with missing values in > 24 samples were eliminated.

#### 2.2.5 Determination of the effect of genetic variant in gene coding sequences

The mutational landscape of the expressed genes in the six tolerant and six sensitive genotypes was investigated using the program SnpEff (Cingolani et al., 2012a). SNP were annotated on their genomic locations and their potential coding effects. The resulting annotated VCF file was filtered using SnpSift (Cingolani et al., 2012b) for homozygous SNP scored as having high or moderate impact in the protein sequence. To determine variants associated with the tolerant or sensitive status of the genotypes, a case-control association analysis with the variant-allele frequencies was done using the SnpSift Case-Control tool. The statistical test used was Fisher’s exact test for the dominant and recessive models. Variants with p-value< 0.001 were considered candidate causal mutations.

#### 2.2.6 Network analysis

We used weighted gene co-expression network analysis (WGCNA) to identify co-expressed gene modules in the dataset (Langfelder and Horvath, 2008, 2012). The normalized expression data of the samples (obtained with the R package Deseq2, Love et al., 2014) was examined using a Euclidean distance-based sample network to detect outliers, with a -2.5 threshold (Horvath, 2011). After outlier elimination, 16 individual samples, representing 4 susceptible cultivars, and 14 individual samples, representing 5 tolerant cultivars were included in the network analysis. Each cultivar was represented by at least 2 samples, with each sample composed of tissue from four seedlings. The analysis was performed using the R package WGCNA (Langfelder and Horvath, 2008, 2012), with signed network type and power 20. The eigengene value of the module in each sample represent the summarized expression of a group of co-expressed genes. We fitted a univariate regression model between the module eigengene and the trait values to identify differentially expressed modules between our tolerant and sensitive samples, using the function lmfit of the R package limma (Ritchie et al., 2015). Modules were considered differentially expressed in the tolerant vs sensitive samples if the Benjamini and Hochberg (1995) adjusted p-value was ≤ 0.001. Modules with high trait significance may represent pathways associated with the expression of the trait. Within modules, genes with high intra-modular connectivity (hub genes) can be considered as the module representative.

#### 2.2.7 Correlating gene expression patterns expression of flumioxazin tolerance

For modules 16, 59, 18, and 65, the relationship between the average module eigengene expression value and the 2-year average PDperc of the cultivars was determined using the cor function in the Stats package (R studio). The Pearson correlation coefficient between those variables was determined for all cultivars included in the network analysis and for those within the tolerant and susceptible groups individually.

#### 2.2.8 Transcription factor binding site motifs search

Analysis of Motif Enrichment software package v.5.5.5 (McLeay and Bailey, 2010) part of the MEME suite (Bailey et al., 2015, available online at https://meme-suite.org), was used to search for enriched motifs in the upstream DNA sequences of the genes present in each of the modules. The sequences were searched against a set of 872 motifs, obtained from the *A. thaliana* database of transcription factors binding sites (O’Malley et al., 2016). For AME to determine which motifs are relatively enriched in one set of sequences, it must use background sequences as a control against which to test for enrichment. A larger set of control sequences allows higher sensitivity. It is recommended to use at least 1000 sequences in the control group if the primary sequence number is< 500. For this analysis, a control set of 2000 genes randomly selected from modules not significantly associated with the tolerant or sensitive status of the cultivars was used to score for enrichment, exceeding the recommended amount. For each gene, the sequence information in FASTA format of the segment 1200 bp upstream of the transcription start site was downloaded from the Phytozome 13 database, reference genome *Phaseolus vulgaris* v.2, using the Biomart tool. The motif enrichment test was Fisher’s exact test (one-tailed).

### 2.3 Candidate genes putative functions

The functional annotations were downloaded from the Phytozome 13 database using the Biomart tool. Putative function, experimental evidence, and phenotype of mutants for the Arabidopsis homologs of the *P. vulgaris* genes were obtained from the TAIR database (*Arabidopsis.org*).

## 3 Results

### 3.1 Environmental conditions

Growing degree days from planting to three weeks later were relatively similar across years (**Supplementary Figure 1A**). Water supply (rainfall plus supplemental irrigation) differed between years (**Supplementary Figure 1B**). The experiment in 2021 had about double the amount of water compared to 2022. In 2021, eight days after planting, a high rainfall event resulted in soil splashing on the expanding leaves of emerged seedlings. This resulted in severe burn on most plants two days later.

### 3.2 Distribution of traits in the association panel

The original (non-transformed) phenotypic values of the genotypes for each trait were evaluated by year to analyze their distribution (**Supplementary Figure 2**). Normality tests failed to reject the assumption of normality for seed weight; PDperc and BPperc were skewed to the right in both years, reflecting the sensitivity of most entries to flumioxazin. For both herbicide tolerance traits, the values in 2021 were lower than those in 2022. The greater water supply in 2021 likely contributed to greater herbicide mobilization and bioavailability, compared to 2022. The transformed phenotypic values were closer to normality, but no transformation resulted in Shapiro-Wilk statistic > 0.05 (**Supplementary Figure 3**).

### 3.3 Pearson’s correlation coefficient of traits across years and ANOVA

**Figure 1:**
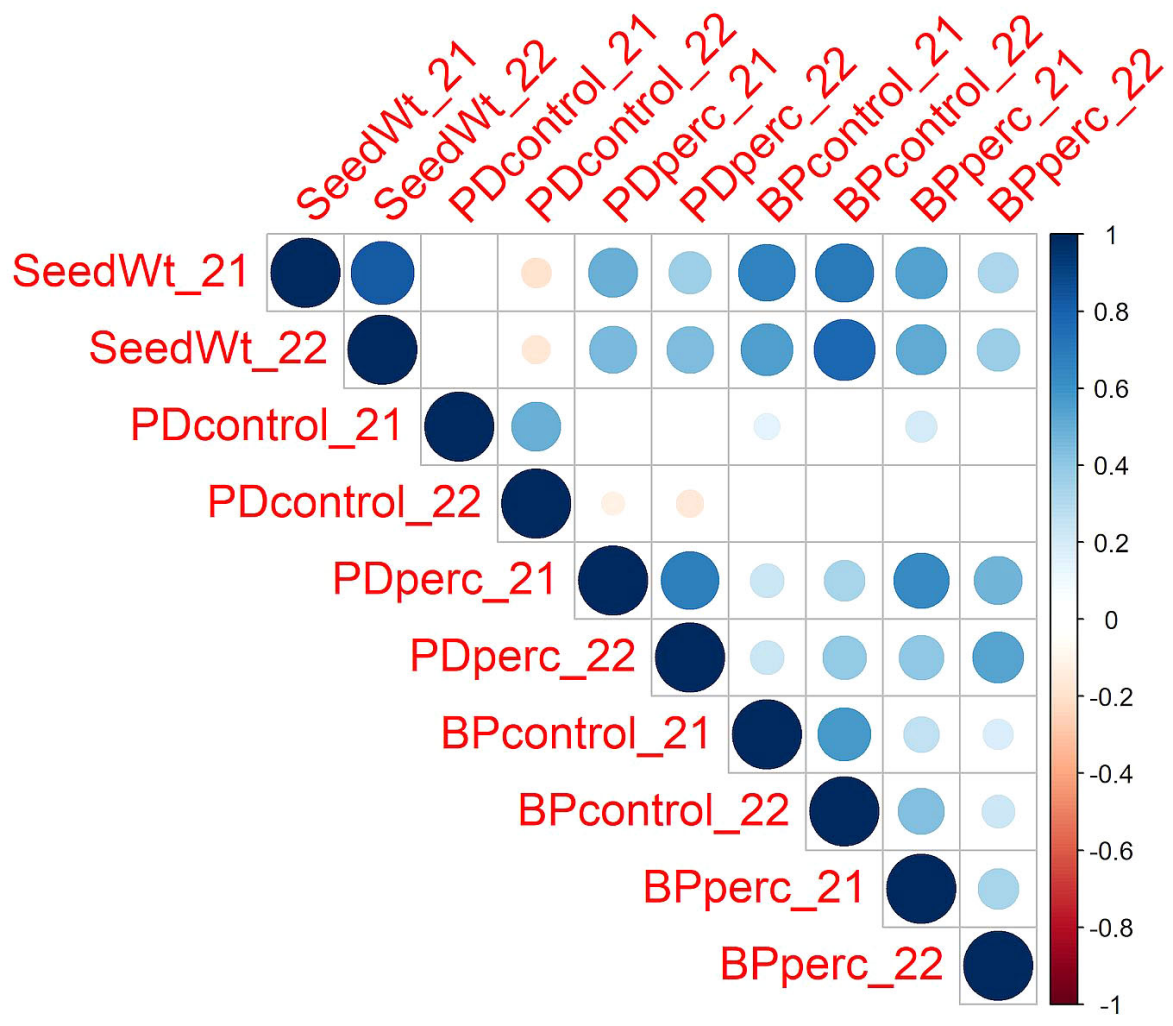
Pearson correlations among traits across the two years of the study. The color and size of the circle depict the strength of the relationship. Only correlations with p ≤0.05 are shown. SeedWt, 100-seed weight in grams of planted lots. PDcontrol, percentage of emerged plants out of 30 planted seeds in the control plots. PDperc, emerged plants in the treated plots as percentage of emerged plots on the control plots. BPcontrol, dry weight per plant in grams in control plots. BPperc, dry weight per plant in the treated plots expressed as percentage of the weight per plant in the control plots.

Despite year-to-year variation, the values of traits were positively correlated across years (**Figure 1**). The strength of the relationship was high for seed weight (*r* = 0.82). Under control conditions, the PD and BP traits in 2021 and 2022 were moderately correlated (*r* = 0.50 and 0.57 for PDcontrol and BPcontrol, respectively). For the tolerance traits, PDperc had a moderately high correlation across years (*r* = 0.69), while BPperc had a lower correlation (*r* = 0.34). It is possible that environment had a greater effect on BPperc than other traits. It is known that the phytotoxicity of flumioxazin is dependent on soil moisture (Sebastian et al., 2016). Wetter growing conditions may have increased injury in partially tolerant genotypes in 2021 compared to 2022. In addition, flumioxazin phytotoxicity is more severe if applied post-emergence, even in crops for which it is registered as preemergent weed control (McNaughton et al., 2014). The high rainfall event in 2021 during emergence of seedlings may have splashed the herbicide onto the leaves, increasing the level of injury and reducing the correlation between years. Analysis of variance confirmed the significant effect of genotype and year in the response of snap bean to flumioxazin (**Table 1**). The significant cultivar-by-environment interaction indicated that cultivars could have different responses across environmental conditions; however, a majority of the most tolerant cultivars were consistent across years, indicating that cultivars with genetic tolerance to flumioxazin are likely to express the trait under different environmental conditions.

**Table 1:** Analysis of variance.

PDperc				
Factors	Sum Sq	DF	F value	Pr(>F)
Cultivar	447750	379	11.04	< 2.2e-16
Year	117459	1	1097.77	< 2.2e-16
Cultivar x Year	100984	376	2.51	< 2.2e-16
Block (Year)	2166	2	10.12	4.30e-05
Residuals	163066	11524		

BPperc				
Factors	Sum Sq	DF	F value	Pr(>F)
Cultivar	418883	379	2.66	< 2.2e-16
Year	309378	1	745.32	< 2.2e-16
Cultivar x Year	207969	375	1.34	1.33e-04
Block (Year)	5281	2	6.36	1.78e-03
Residuals	596489	1437		

Significance of main effects of snap bean cultivar, year and block, and their interactions on crop response in the field experiment. PDperc, Plant density in flumioxazin treated plots as percentage of non-treated plots. BPperc, Dry biomass per plant in flumioxazin treated plots as percentage of non-treated plots.

### 3.4 Correlation among traits

Pearson analysis of correlation among traits revealed a significant positive correlation between seed weight and both PDperc and BPperc (*r* = 0.49 and *r* = 0.57, respectively), while it was negatively correlated with PD under control conditions (*r* = -0.14). The correlation between PDperc and BPperc was positive (*r* = 0.67). In contrast, PDcontrol and BPcontrol were not correlated. This suggests that in the control treatment, genetic factors that promote germination and growth are mainly unrelated to each other, whereas in the herbicide treatment, shared genetic factors may influence both germination and growth. The positive correlations observed between BPperc and BPcontrol in both years indicate that seedling vigor contributes to flumioxazin tolerance.

### 3.5 Response of snap bean cultivars to flumioxazin

There were varying degrees of sensitivity to flumioxazin among genotypes, affecting both plant density and biomass. Some genotypes were highly tolerant, some showed intermediate response, and the majority were sensitive. The ten most tolerant and ten most sensitive genotypes are shown in **Table 2**. It is noteworthy that most accessions with high flumioxazin tolerance belong to the market class “Romano”, characterized by a flat pod phenotype, and seed weight above the panel average. In the full panel, the means of the traits PDperc and BPperc of the cultivars classified as “Romano” were significantly different from the combined mean of the other classes, based on the Welch two sample t-test (*p* value = ≤3.40e-06).

**Table 2:** List of snap bean genotypes in the SNAP diversity panel most tolerant and more sensitive to flumioxazin based on the tolerance evaluated as the average plant density and biomass per plant, in a field experiment near Urbana, IL in 2021 and 2022.

Genotype	SnapID	PI no.	Type	Seed Wt	Pod Shape	PDperc	BPperc
Tolerant							
Trend*	SnAP356	NA	Romano	62.54	Flat	81.3	86.1
Bush Romano 350	SnAP056	PI 538770	Romano	45.805	Flat	62.5	98.1
Roma II	SnAP279	PI 549997	Romano	47.29	Flat	75.9	68.2
Romano Purpiat	SnAP282	NA	Romano	37.285	Flat	54.9	89.2
DMC 06–01	SnAP095	PI 560313	Romano	45.35	Flat	73.6	63.5
Jumbo	SnAP187	PI 550044	Romano	52.635	Flat	63.5	65.3
Moncayo	SnAP227	PI 598219	Romano	37.635	Flat	78.7	50.7
Romano 118	SnAP280	NA	Romano	47.685	Flat	63.3	59.9
Bountiful	SnAP045	PI 598998	Fresh market	45.81	Flat	70.8	53.0
DMC 06–39	SnAP096	PI 560314	Romano	60.625	Flat	57.4	56.1
Sensitive							
Redon	SnAP269	PI 639240	Processing	10.235	Round	2.0	7.5
Lakeland	SnAP200	PI 549978	Processing	31.711	Round	2.7	5.7
Wax 216	SnAP369	PI 550408	wax - dual	31.935	Round	2.4	5.5
DMC 04–01	SnAP087	PI 564075	Processing	36.72	Round	2.8	4.1
Booster	SnAP044	NA	Processing	10.315	Round	1.7	6.5
Polder	SnAP258	PI 603217	Processing	20.545	Round	1.8	5.8
Juliet	SnAP186	NA	Fresh market	17.465	Craseback	0.6	5.6
Landmark	SnAP202	NA	Fresh market	36.05	Craseback	1.0	1.0
Isar	SnAP180	NA	Fresh market	13.685	Round	1.6	0.5
Selecta	SnAP294	NA	Processing	11.37	Round	0.0	0.0

*Cultivar Trend present in the SnAP does not correspond to the publicly available germplasm collection entry PI 550128.

SnapID, Identification number given in the SnAP. PI no., Plant identification number in the U.S. National Plant Germplasm System. PDperc, Plant density in flumioxazin treated plots as percentage of non-treated plots. BPperc, Dry biomass per plant in flumioxazin treated plots as percentage of non-treated plots. NA, no information available

### 3.6 Trait heritability

High broad-sense heritability (*H* ^2^ = 0.79) was observed in PDperc (**Table 3**). In a highly inbred population such as SnAP, the dominance effects do not contribute to the phenotype of the lines (Falconer and Mackay, 1996). Therefore, the broad-sense heritability should approximate the narrow-sense heritability. Nonetheless, the narrow-sense heritability was moderate (*h* ^2^ = 0.57). Similarly, the broad-sense heritability estimate for BPperc was moderate (*H* ^2^ = 0.49, **Table 3**), and the narrow-sense heritability was low (*h* ^2^ = 0.19). These results indicated that genetic improvement of flumioxazin tolerance would be more successful in increasing the germination percentages under preemergence application of the herbicide than the biomass accumulation after emergence. The lower heritability of BPperc could reflect the lower level of tolerance to the post-emergence exposure to flumioxazin, which resulted in extensive damage to seedlings in 2021.

**Table 3:** Broad and narrow sense heritability of flumioxazin tolerance traits in snap bean as measured in a field experiment near Urbana, IL, in 2021 and 2022.

Trait	Broad-sense (*H* ^2^)	Narrow-sense (*h* ^2^)	95% CI of *h* ^2^
PDperc	0.79	0.57	0.51–0.63
BPperc	0.49	0.19	0.13–0.25

PDperc, Plant density in flumioxazin treated plots as percentage of non-treated plots. BPperc, Dry biomass per plant in flumioxazin treated plots as percentage of non-treated plots.

### 3.7 Genome-wide association studies

#### 3.7.1 Significant genomic locations for plant response to flumioxazin

Analysis of the pairwise *r^2^* between marker demonstrates that LD decays rapidly between 0 and 2 Mbp of distance, reaching< 0.5 at 162 Kbp of distance between markers in average (**Supplementary Figure 4**). As the extent of LD determines the required number of SNP markers and the mapping resolution (Flint-Garcia et al., 2003), it would be expected that a marker density of at least 6.2 SNPs/Mbp would be needed to efficiently detect associated loci.

**Figure 2:**
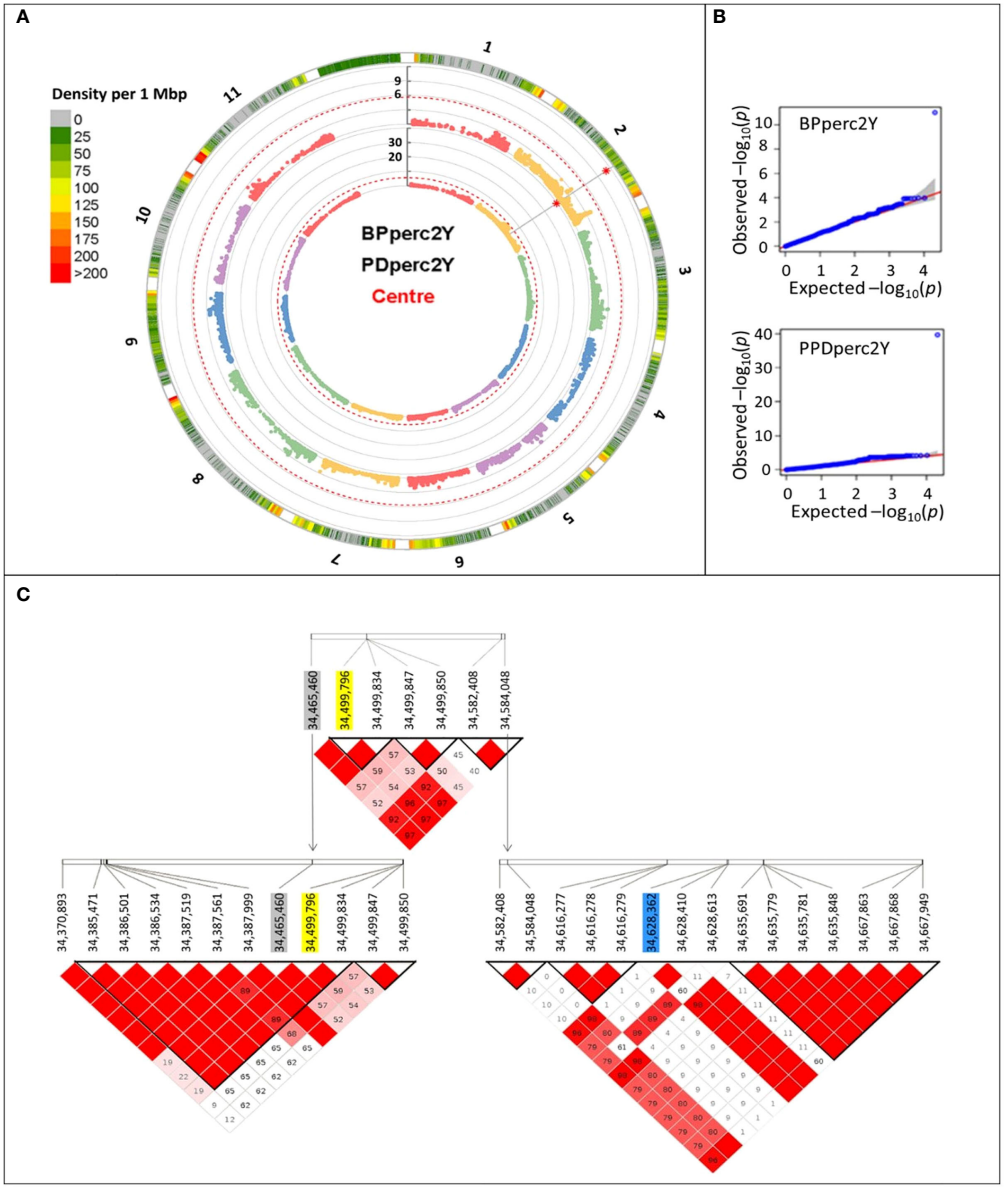
Manhattan plots summarizing the results of the genome-wide association analyses for the two-year average of flumioxazin tolerance traits for the 377 cultivars of the *Phaseolus vulgaris* snap bean association panel grown at Urbana, IL, USA, in 2021 and 2022. **(A)** Circular Manhattan plots of the MLMM analyses. Rings from outer boundary to center: Chromosome number, SNP marker density, significance of each marker association with BPperc trait, significance of each marker association with PDperc trait. The vertical axis shows the -log10 of the p-value of the association. The red dashed line represents the significance threshold. Red asterisk indicates the position of significant SNP markers. **(B)** Quantile-quantile plots depicting the observed (Y-axis) and expected (X-axis) -log10 of the p-value. **(C)** Graphical representation of the linkage disequilibrium within the overlapping haploblocks. Significantly associated SNP markers are shaded. Yellow: Detected in year 2022 and joint-years analysis. Grey: Detected in 2021 analysis. Blue: Detected in 2022 analysis.

To reduce the environmental influence, GWAS analysis was performed using the BLUP of the traits; therefore, most of the remaining phenotypic variation is expected to be due to genotype differences. For both PDperc and BPperc, a genomic region delimited by three linked markers was detected in joint years and individual years analyses (**Table 4**; **Figure 2**). The chromosomal region in which the significant SNPs are located has a marker density of 50–75 SNP/Mbp (**Figure 2A**). Examination of the quantile-quantile plots reveal that most of the p-values follow a uniform distribution, apart from a few SNP with very low *p*-values, likely indicating that the model successfully accounted for covariance and population structure. The marker density in the significant region Depending on the year and trait, one of three SNPs was significantly associated with the tolerance traits within a 162.9 kbp segment in chromosome 2. Two of the SNPs are in the overlap region of two LD blocks (34,370,893 to 34,499,850 bp, and 34,465,460 to 34,584,048 bp) while the third SNP belongs to an LD block from 34,582,408 to 34,667,949. The significant SNPs in this region explained a high proportion of the PDperc phenotypic variance (PV) in the joint-year analysis (80.10%). For BPperc, the SNP explained 34.43% of the PV.

**Table 4:** Genomic regions associated with plant density percentage (PDperc) and biomass per plant percentage (BPperc) identified under the MLMM model.

Marker	Chr	Pos	MAF	Adj. *p-*value	PV (%)
Joint 2021–2022 analysis					
PDperc 2Y transformed					
S02_34499796	2	34,499,796	0.22	4.81E-36	80.10
BPperc 2Y transformed					
S02_34499796	2	34,499,796	0.22	2.05E-07	34.43
2021 analysis					
PDperc 21 transformed					
S02_34465460	2	34,465,460	0.21	1.18E-13	55.18
BPperc 21 transformed					
S02_34465460	2	34,465,460	0.21	8.04E-07	42.15
2022 analysis					
PDperc 22 transformed					
S02_34499796	2	34,499,796	0.22	1.51E-31	74.27
BPperc 22 transformed					
S02_34628362	2	34,628,362	0.22	8.59E-05	27.73

PV, phenotypic variance explained.

#### 3.7.2 Genomic prediction

GAPIT estimated genomic breeding values as well as their prediction accuracy. The PDperc estimates under the gBLUP model using the full panel were highly correlated with the observed 2-year average phenotypic values for PDperc (*r* = 0.96). The accuracy of genome prediction was investigated through cross-validation. The average value of the correlation in 1000 runs was *r* = 0.79 with a standard deviation of 0.01 for the reference set, and *r* = 0.52 with a standard deviation of 0.10 for the inference set. For BPperc, the correlation between the predicted and observed values was 0.60. The accuracy of the prediction was low, with the 1000 runs average of *r* = 0.58 and *r* = 0.20 for the reference and inference set, respectively. Cultivars with predicted PDperc values > 40% per market class are presented in **Table 5**. Such cultivars are a source of tolerance alleles for snap bean breeding programs.

**Table 5:** SNP bean cultivars with predicted PDperc values > 40% across market class and sieve size.

Cultivar name	SnapID	Predicted PDperc	Observed PDperc	Seed Wt.	Allelic status	Type	Sieve size
Bountiful	SnAP045	62.16	70.82	45.81	GG	Fresh market	Flat
Kentucky Wonder Bush	SnAP189	47.76	53.02	46.49	GG	Fresh market	4–5
Burpee’s Stringless	SnAP052	45.69	49.40	41.94	GG	Fresh market	5
Climbing French	SnAP075	48.63	45.75	53.36	GG	Fresh market pole	Flat
Magnum	SnAP209	40.13	43.22	46.75	GG	KY flat	Flat
Green Arrow	SnAP161	40.52	44.90	25.08	GG	NA	3–4
Bush Blue Lake Supreme	SnAP054	40.97	49.30	46.41	GG	Processing	4–5
Trend	SnAP356	73.27	81.27	62.54	GG	Romano	Flat
Moncayo	SnAP227	68.99	78.72	37.64	GG	Romano	Flat
Romano 118	SnAP280	63.16	63.33	47.69	GG	Romano	Flat
Roma II	SnAP279	68.46	75.90	47.29	GG	Romano	Flat
Bush Romano 350	SnAP056	62.35	62.50	45.81	GG	Romano	Flat
DMC 06–01	SnAP095	65.66	73.55	45.35	GG	Romano	Flat
Jumbo	SnAP187	64.51	63.52	52.64	GG	Romano	Flat
Romano Purpiat	SnAP282	52.08	54.87	37.29	GG	Romano	Flat
Gina	SnAP149	52.70	56.18	48.47	GG	Romano	Flat
DMC 06–39	SnAP096	61.42	57.43	60.63	GG	Romano	Flat
Navarro	SnAP230	42.68	49.27	41.41	AA	Romano	Flat
Wax Romano 82264	SnAP370	42.42	44.37	41.72	AA	Romano	Flat
Bush Romano 71	SnAP058	48.22	38.48	45.50	GG	Romano	Flat
Roma	SnAP278	40.87	43.98	39.31	GG	Romano	Flat

Predicted and observed values refer to the plant density in flumioxazin treated plots as percentage of non-treated plots. Allelic status refers to SNP marker S02_34499796. NA, no information available.

#### 3.7.3 Influence of seed weight and allelic status at the significant genomic interval on flumioxazin tolerance

**Figure 3:**
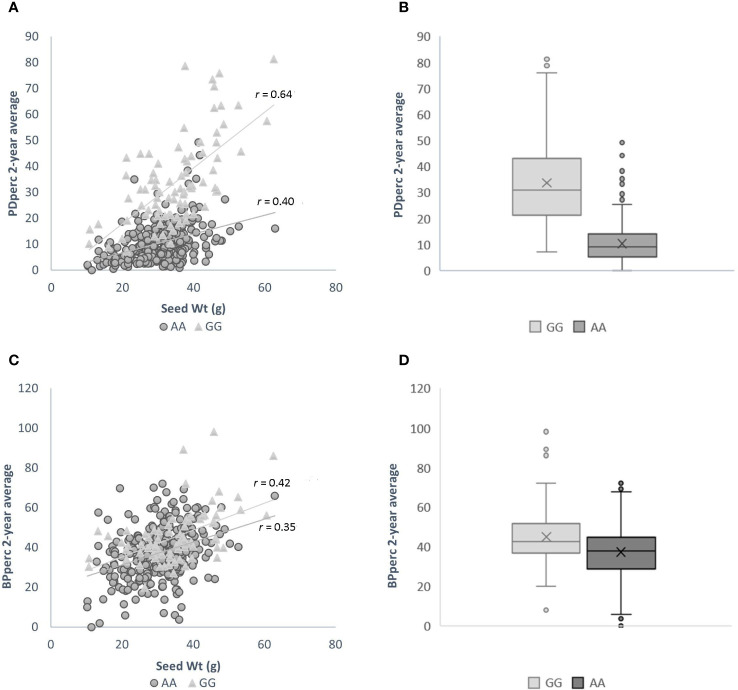
Effect of genotype at SNP marker S02_34499796 and seed size on the response to flumioxazin of the cultivars in the snap bean association panel. **(A)** Relationship between seed weight and plant density percentage for cultivars with positive effect allele (light grey triangles), and negative effect allele (dark grey circles). **(B)** Box-and-Whisker plots showing the distribution of PDperc values in cultivars of the SnAP carrying the positive (light grey fill) and negative (dark grey fill) effect alleles. Boxes represent median and interquartile range, the mean is depicted by an x. **(C)** Relationship between seed weight and biomass per plant percentage for cultivars with positive effect allele (light grey triangles), and negative effect allele (dark grey circles). **(D)** Box-and-Whisker plots showing the distribution of BPperc values in cultivars of the SnAP carrying the positive (light grey fill) and negative (dark grey fill) effect alleles. Boxes represent median and interquartile range, the mean is depicted by an x.

The influence of seed weight and allelic status is evident in the scatter plots of 2-year average PDper by seed weight when the population is divided by the allelic status at the significant SNP. In agreement with correlation analysis between the traits’ BLUP, there is a positive correlation between seed weight and the average of observed PDperc; however, the strength of the correlation varies depending on the allelic status of the cultivars at marker S02_34499796, the SNP marker most significantly associated with the tolerance. Across the whole panel, the correlation of seed weight with PDperc was *r* = 0.49, while the correlation between the same variables using only the population of entries with the positive effect allele was *r* = 0.64, and the correlation of the entries with the negative effect allele was *r* = 0.40 (**Figure 3A**). The effect of the allelic status can be visualized in the box plots of all the cultivars with contrasting alleles. The difference between the means of the two groups is 23.64 percentage points, which is significant at α ≤ 0.05 (**Figure 3B**). It is evident from the graph that the combination of heavier seed (> 40 g per 100-seed) and presenting the favorable (GG) version of the significant marker is likely to confer commercially viable tolerance to flumioxazin at the 2X recommended rate. For the BPperc trait, the correlation with seed weight was moderate for the set of the cultivars with the positive effect allele (*r* = 0.42) and low for the cultivars with the negative effect allele (*r* = 0.35) (**Figure 3C**). The mean difference between the group of cultivars with the favorable allele and the ones without is 8.13 percentage points (**Figure 3D**). Although the difference between the groups is relatively small, it is significant at α ≤ 0.05.

#### 3.7.4 Gene models in the haploblock interval

Genes within the region delimited by the start of haploblock of markers S02_34465460 and S02_34499796, and the end of haploblock of marker S02_34628362, were further investigated. Tolerance to PPO-inhibitors such as flumioxazin can be due to TSR, in which changes in the PPO enzymes prevent the binding of the herbicide molecule. Alternatively, NTSR mechanisms confer tolerance via lower absorption and mobilization of the herbicide, degradation, sequestration and excretion of the herbicide molecule, or amelioration of the damage caused by the free radicals. Genes with homology to genes with validated function in those processes and present in the haploblock of the associated SNP could be candidate genes for tolerance to flumioxazin.

The single region detected in the GWAS analysis could suggest TSR. However, the genes encoding the PPO enzymes in the *P. vulgaris* reference genome version 2 are located in Chr01: 33,098,528–33,103,479 bp and Chr07: 18,072,151–18,094,811 bp for PPO1 and PPO2, respectively. Therefore, TSR is not responsible for the tolerance observed in the SnAP.

Nineteen gene models are located in the haploblocks for the significantly associated SNPs (**Table 6**). The putative functions and functional domains of the genes found in the interval were compared to those in the models for xenobiotic detoxification and oxidative stress tolerance from Edwards et al. (2011); Cavé-Radet et al. (2020), and Rigon et al. (2020), to identify genes with possible roles in the mechanisms of tolerance. In previous work with another PPO-inhibiting herbicide, sulfentrazone, genes encoding cytochrome P450 enzymes, possibly involved in herbicide degradation, and ABC transporters, possibly involved in xenobiotic sequestration, were found in the intervals (Saballos et al., 2022). Surprisingly, no genes coding for proteins with homology to proteins directly implicated in herbicide tolerance are located in the interval, opening the possibility that novel mechanisms of NTSR are controlling the tolerance to flumioxazin in snap bean. Three predicted proteins designated as oxidoreductases are present. Oxidoreductases could be part of the processes that maintain the cell’s redox status and ameliorate oxidative stress. Three predicted proteins in the interval contain DNA-binding domains, which could implicate them in the regulation of transcription of other genes involved in processes that ultimately result in the snap bean response to flumioxazin.

**Table 6:** Gene models located in the chromosome 02 region associated with flumioxazin tolerance and their predicted functions.

Gene Name	Gene Start (bp)	Gene End (bp)	Description	GO Description
Phvul.002G183200	34,383,753	34,385,842	NADH dehydrogenase transmembrane subunit	Oxidoreductase activity
Phvul.002G183300	34,386,082	34,392,004	Uncharacterized conserved protein	
Phvul.002G183400	34,403,190	34,404,179	AGAMOUS-LIKE 82	DNA binding
Phvul.002G183450	34,407,410	34,410,148		NAD+ ADP-ribosyltransferase activity
Phvul.002G183500	34,414,042	34,418,557	Ubiquitin-conjugating enzyme E2	
Phvul.002G183600	34,419,868	34,422,386	Protein of unknown function	
Phvul.002G183700	34,421,845	34,422,455		
Phvul.002G183800	34,426,045	34,427,997	PPR repeat family (PPR_2)	Protein binding
Phvul.002G183900	34,428,903	34,439,086	2OG-FE II oxygenase family protein	oxidoreductase activity
Phvul.002G184000	34,446,710	34,447,897	Clathrin assembly protein	Phospholipid binding
Phvul.002G184100	34,465,644	34,467,876	Unknown	
Phvul.002G184200	34,468,080	34,481,795	Solute carrier family 39 (zinc transporter)	Membrane
Phvul.002G184300	34,495,833	34,497,370	Flavonone synthase	Catalytic activity
Phvul.002G184400	34,503,835	34,506,648	2-oxoglutarate and FE(II)-dependent oxygenase	oxidoreductase activity
Phvul.002G184500	34,518,080	34,519,970	Basic pentacysteine1-related protein	Nucleic acid binding
Phvul.002G184700	34,552,415	34,555,140	Serine-threonine-protein kinase	Nucleic acid binding
Phvul.002G184800	34,581,234	34,585,239	Serine-threonine-protein kinase BRI1-LIKE 2	protein kinase activity
Phvul.002G184900	34,607,469	34,617,874	Transmembrane 9 superfamily protein	Integral component of membrane
Phvul.002G185000	34,613,244	34,614,758	Unknown	

### 3.8 RNA sequence analyses

#### 3.8.1 RNA sequencing

Gene expression was measured by sequencing RNA from germinating seedlings of 12 snap bean cultivars. Tolerance and sensitivity were represented each by 6 cultivars and 4 biological replicates, except for two cultivars that had 3 replicates each. For each sample, at least 50 million filtered reads were obtained and the alignment rate to the *P. vulgaris* genome was greater than 89% (**Supplementary Table 2**).

#### 3.8.2 Differential expression and GO term enrichment of the flumioxazin tolerant transcriptome

**Figure 4:**
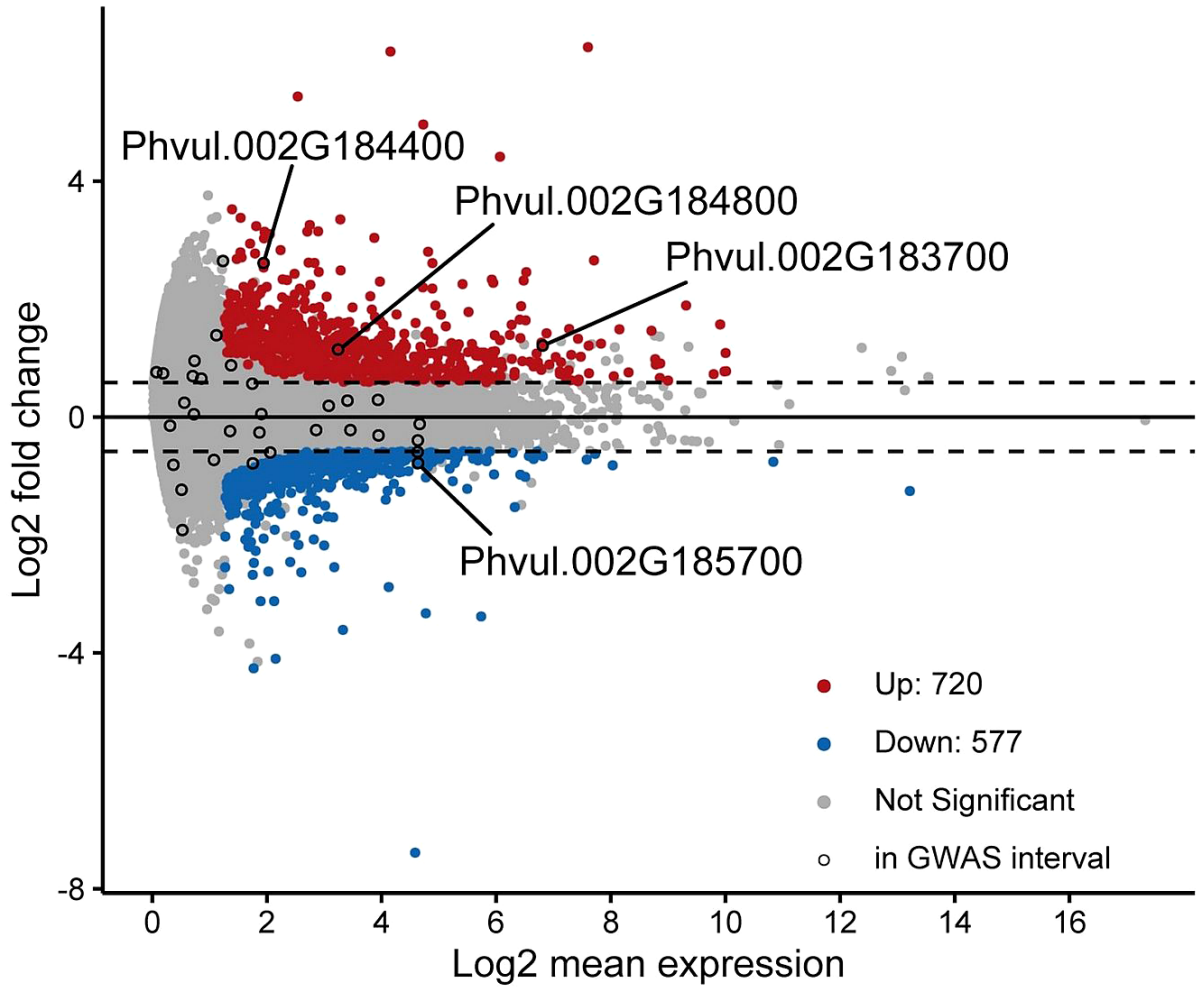
MA-Plot of gene expression comparison between tolerant and susceptible cultivars. Differentially expressed genes were those with a fold-change ≥ 1.5 and adjusted p-value ≤ 0.1 and are represented by red and blue points for those that are upregulated and downregulated, respectively. Black circles indicate expressed genes that fall within the GWAS interval. Four genes that are in the GWAS interval and differentially expressed at the above cutoffs are labeled.

Differentially expressed genes between the tolerant and sensitive cultivars were identified with cutoffs of fold-change ≥ 1.5 and adjusted p-value ≤ 0.1. A total of 1297 genes were identified as differentially expressed with 720 genes upregulated and 577 genes downregulated in the tolerant versus sensitive groups of cultivars (**Figure 4**).

All differentially expressed genes were functionally classified by Gene Ontology term enrichment analyses (**Supplementary Figures 5, 6**). Of the 1297 DEG, 757 genes were associated with GO terms. Upregulated genes were enriched for GO terms related to biological processes involved in protein-DNA assembly, cellular components involved in chromosome organization, and molecular function in oxidoreductase activity. Down-regulated genes were enriched in multiple terms, most notably for cellular components related to cellular and organelle membrane, Golgi apparatus and vesicles; molecular function of protein binding, and ligase and helicase activity; and biological processes related to protein localization.

#### 3.8.3 Effect of genetic variants in gene coding sequences

We used the sequence information obtained from the RNA sequencing of tolerant and sensitive cultivars to investigate sequence variation of the genes expressed during germination in the GWAS interval and surrounding 1000 kbp area. Of the 23 gene models located within that region, 20 were expressed in all cultivars. Phvul.002G183400, Phvul.002G183450, and Phvul.002G184000 did not produce reads in any sample, agreeing with the data available at the bean expression atlas (O’Rourke et al., 2014) in which those genes had very low levels of expression in all tissues evaluated. It is unlikely those genes play a significant role in flumioxazin tolerance. Of the expressed genes, 12 had variants that were significantly associated with the phenotype and that would likely result in moderate to high impact mutations in the protein products (**Table 7**).

**Table 7:** Sequence variation associated with tolerance phenotype in genes expressed during germination in 12 snap bean cultivars.

Position Chr02 (bp)	Effect	Change	Adj. *p*-value	Gene model
34,386,501.00	Stop lost	c.1780T>C|p.Ter594G extension	1.14E-25	Phvul.002G183300
34,386,534.00	Missense	c.1747A>G|p.Asn583Asp	1.14E-25	Phvul.002G183300
34,387,606.00	Missense	c.1304C>T|p.Pro435Leu	1.14E-25	Phvul.002G183300
34,389,673.00	Missense	c.775C>G|p.Pro259Ala	1.14E-25	Phvul.002G183300
34,391,800.00	Missense	c.13C>A|p.His5Asn	4.63E-25	Phvul.002G183300
34,414,612.00	Missense	c.23A>T|p.Gln8Leu	1.55E-17	Phvul.002G183500
34,422,373.00	Missense	c.179A>G|p.Gln60Arg	3.03E-08	Phvul.002G183700
34,426,282.00	Missense	c.7T>G|p.Trp3Gly	1.42E-14	Phvul.002G183800
34,426,288.00	Missense	c.13G>A|p.Asp5Asn	5.51E-15	Phvul.002G183800
34,426,901.00	Missense	c.626C>G|p.Ala209Gly	2.48E-14	Phvul.002G183800
34,429,049.00	Missense	c.961T>C|p.Ser321Pro	5.79E-08	Phvul.002G183900
34,438,914.00	Missense	c.124C>G|p.Leu42Val	8.55E-04	Phvul.002G183900
34,481,437.00	Missense	c.54G>C|p.Leu18Phe	4.63E-25	Phvul.002G184200
34,481,457.00	Missense	c.34T>C|p.Ser12Pro	4.63E-25	Phvul.002G184200
34,496,654.00	Missense	c.517T>A|p.Leu173Met	2.47E-12	Phvul.002G184300
34,554,524.00	Missense	c.787G>A|p.Val263Ile	4.05E-21	Phvul.002G184700
34,582,695.00	Missense	c.2293C>G|p.Leu765Val	1.81E-15	Phvul.002G184800
34,584,048.00	Missense	c.940G>A|p.Ala314Thr	2.41E-24	Phvul.002G184800
34,614,063.00	Start lost	c.2T>A|p.Met1?	1.92E-11	Phvul.002G185000
34,614,365.00	Missense	c.304T>C|p.Ser102Pro	3.01E-11	Phvul.002G185000
34,616,880.00	Missense	c.302T>C|p.Val101Ala	2.43E-27	Phvul.002G184900
34,648,219.00	Missense	c.1742T>G|p.Phe581Cys	6.94E-24	Phvul.002G185150

#### 3.8.4 Network analysis and GO term enrichment of phenotype associated modules

We used weighted gene co-expression network analysis (WGCNA) to identify co-expressed gene modules across 30 samples representing 9 cultivars of snap bean. The cultivars included in the experiment represented contrasting responses to flumioxazin within a narrow range of seed weight. In addition, the cultivars classified as tolerant carry the positive effect allele of the SNP markers in the GWAS identified region. This region explains most of the variance; therefore, it is likely that all the tolerant cultivars share the same tolerance mechanism. Twelve gene modules were significantly associated with the tolerance status with an adjusted p-value ≤ 0.001. The correlation between the average eigengene value of the cultivars for the each of the significant modules and their 2-year average PDperc values ranged from *r* = -0.98 to -0.64 and *r* =0.99 to 0.65 for the modules downregulated and upregulated in the tolerant cultivars, respectively. Modules with |*r*| > 0.75 were further investigated. Two of the modules are composed of genes whose expression was upregulated in the tolerant samples and two are downregulated (**Supplementary Figure 7**; **Supplementary Tables 3–6**). Only modules 16 and 18 showed consistent expression patterns in all samples within cultivars of same tolerance status, with |*r*| > 0.98. Upregulated modules included 16 and 59. For module 16, the correlation coefficient of the average eigengene value of all the cultivars and their PDperc values was *r* = 0.99. The correlation coefficients of those variables within the cultivars with tolerant or sensitive response were not significant at α ≤ 0.05 (**Supplementary Figure 8A**). Module 16 contains 380 gene models, of which 206 have associated GO terms. Two GO terms are enriched in the module, both related to oxidoreductase activity. This includes six predicted cytochrome P450 enzymes whose homologs are involved in flavonoid, phytoalexin, suberin and wax biosynthesis and are upregulated in response to biotic and abiotic stresses in other species. Notably, Phvul.010G013100, coding for a homolog of maize cytochrome P450 enzyme CYP81A9 is present in this module. CYP81A9, synonym Nsf1, is responsible for the detoxification of a wide range of herbicides in maize (Nordby et al., 2008). The soybean homolog to Phvul.010G013100, *CYP81E22* has been identified as the causative gene for the sensitivity of soybean to the herbicide bentazon (Kato et al., 2020). Oxidoreductases, oxygenases and peroxidases are also present in this module. Enzymes in those classes are thought to be involved in ROS stress amelioration and maintenance of the redox status of the cell (Dumanović et al., 2021). Gene models Phvul.006G075800 and Phvul.006G075900 are similar to *Medicago truncatula RIP1*. The peroxidase enzyme encoded by this gene functions on the removal of toxic reductants, and it is induced in response to colonization by symbiotic *Rhizobium* bacteria, pathogen attack and oxidative stress (The UniProt Consortium, 2022; accession number Q40372). Gene model Phvul.002G206900 is annotated as brassinosteroid insensitive 1-associated receptor kinase 1 (BAK1). In Arabidopsis, BAK1 is a multifunctional protein involved in promotion of seedling growth (Ladwig et al., 2015) and control of cell-death. It positively regulates the brassinosteroid (BR)-dependent plant growth pathway and negatively regulates the BR- independent cell-death pathway (He et al., 2007). Other genes in module 16 code for proteins with functions related to the cell wall, including five genes coding for phenylpropanoid biosynthesis enzymes likely involved in lignin synthesis, and three pectinesterase enzymes likely involved in modification of cell walls.

For module 59, the overall correlation coefficient was *r* = 0.80. The correlation coefficient between the eigengene value and the PDperc of the cultivars within response status tolerant was not significant. The eigengene value and the PDperc had a high correlation coefficient (*r* = 0.96) in the cultivars within response status sensitive (**Supplementary Figure 8B**). This could indicate that in the absence of the favorable allele, other factor(s) may be influencing the expression of the genes in the module, providing increased tolerance. Module 59 contains 61 gene models, of which 42 have associated GO terms. Enriched GO terms for carbohydrate metabolic processes were detected in module 59. As with module 16, multiple genes with homology to genes coding for enzymes implicated in cell wall biogenesis and modification are present, including cellulose and phenylpropanoid biosynthesis.

Down regulated modules included modules 18 and 65. For module 18, the overall correlation coefficient was *r* = -0.98. The correlation coefficients of the cultivars within response status were not significant (**Supplementary Figure 8C**). Module18 is composed of 292 gene models, of which 162 have associated GO terms. Overrepresented terms include those processes related to programmed cell death, immune response, pathogenesis, and lipid modifications. Multiple nucleotide-binding site leucine-rich repeat (NBS-LRR) apoptotic ATPases are present in this module. Homologs to these genes in Arabidopsis and soybean have been identified as possible disease resistance proteins through the hypersensitive response (Ashfield et al., 2004; Bezerra-Neto et al., 2020). The hub gene in this module, Phvul.004G154900, is a protein kinase superfamily protein, similar to leaf rust 10 disease-resistance locus receptor- like protein kinase-like2 of *A. thaliana*. For module 65, the overall correlation coefficient was *r* = -0.89. The correlation coefficients of the cultivars within response status were not significant (**Supplementary Figure 8D**). Module 65 is composed of 63 gene models, of which 35 have associated GO terms. Like module 18, module 65 has over-representation of GO terms related to immune response and programmed cell death. The hub gene, Phvul.002G323704, is a leucine-rich repeat protein, containing the NB-ARC domain, a novel signaling motif found in bacteria and eukaryotes, shared by plant resistance gene products and regulators of cell death.

#### 3.8.5 Transcriptional regulatory elements in the flumioxazin tolerance-associated genomic interval

While the network analysis offers insight into the genes and biological processes likely involved in flumioxazin tolerance in snap bean, the relationship between them and the genomic interval identified by GWAS remains to be explained. One possibility is the presence of transcription factors (TF) that act in the cis-regulatory elements of the genes. To investigate this possibility, we searched for TF in the set of genes in or near the GWAS interval. The genes were selected by three conditions: expressed in the snap bean cultivars at germination, located in the associated interval or 100Kb upstream or downstream of it, and presenting sequence variations of moderate or high effect significantly associated with the phenotypes (*p* value< 0.05). Two expressed transcription factors are located in the interval: Phvul.002G184500, a BBR-BPC family TF, and Phvul.002G184700, a C2H2 family TF. Only Phvul.002G184700 presents a sequence variation associated with the phenotype of the cultivars, and this variation is predicted to cause a Val263Ile substitution. C2H2 are one of the largest families of eukaryotic TF. In plants, increasing evidence has indicated that C2H2-type zinc finger proteins play important roles in abiotic and biotic stress resistance (Kiełbowicz-Matuk, 2012; Han et al., 2020).

#### 3.8.6 Enriched motifs in upstream sequence of genes in significantly associated modules

We investigated the molecular mechanisms of transcriptional regulation of a set of genes belonging to modules associated with the tolerance status of the tested cultivars. Under the hypothesis of co-regulation, it is expected that the genes belonging to a module would share binding sites for the transcription factors regulating their expression. Module 16, the most significantly associated module, which was upregulated in the tolerant entries, was enriched in a motif corresponding to the binding site for Arabidopsis C2H2 family transcription factor AT1G27730 (Salt tolerance zinc finger 10), involved in salt tolerance and response to photooxidative stress (Mittler et al., 2006). The motif is present in the upstream sequence of 54.33% of the genes in the module, including Phvul.010G013100 (**Table 8**). Upregulated Module 59 is enriched for a motif corresponding to the binding site of AT1G73730, ethylene-insensitive3-like 3 TF, present in 14.75% of the gene’s upstream sequences.

**Table 8:** Putative transcription factor binding motifs enriched in the 1200 bp fragment upstream of the transcription start site for the genes in modules 16, 59, and 18.

Module	Motif ID	Consensus motif	*p*-value	Adj. *p*-value	No. seq	TP	%TP	FP	%FP
**16**	ABI3VP1_tnt.AT5G18090_col_a_m1	RRWGATGAADMDDAD	5.89E-12	4.42E-09	381	14	3.67	0	0
	C2H2_tnt.STZ_col_m1	CACTNHCACTN	9.25E-08	9.34E-05	381	207	54.33	794	39.7
	ABI3VP1_tnt.AT5G25475_col_a_m1	CAAGCA	5.79E-07	3.24E-04	381	126	33.07	422	21.1
	MADS_tnt.AGL42_col_a_m1	CATCATY	2.12E-06	2.32E-03	381	130	34.12	452	22.6
	C2C2gata_tnt.GATA12_col_a_m1	RATYYAGATCTRA	1.74E-05	3.55E-03	381	46	12.07	114	5.7
**59**	EIL_tnt.EIL3_col_b_m1	NYGTCYAGRTTCAWWDWADHT	1.53E-05	2.30E-03	61	9	14.75	44	2.2
**18**	C3H_tnt.TZF9_col_a_m1	TCAACA	6.55E-09	8.41E-06	290	100	34.48	380	19
	C3H_tnt.U2AF35B_col_a_m1	TTGTTGA	3.20E-07	3.32E-04	290	89	30.69	351	17.55
	ABI3VP1_tnt.AT5G18090_col_a_m1	RRWGATGAADMDDAD	3.94E-06	2.74E-03	290	6	2.07	0	0
	GRF_tnt.GRF9_colamp_a_m1	TGTCAGAA	1.25E-05	4.42E-03	290	71	24.48	284	14.2
	G2like_tnt.AT4G37180_col_a_m1	HARAAGATTCY	1.47E-05	5.21E-03	290	70	24.14	280	14

No. seq, number of genes present in the module. TP, true positive, number of sequences presenting the motif. %TP, percentage of TP in the module. FP, number of sequences presenting the motive out of the 2000 sequences in the control set. %FP, percentage of FP in the control set.

In downregulated modules, module 18 presented enrichment in the motif corresponding to the binding site of AT5G58620, tandem zinc finger protein 9, involved in control of defense gene expression (Maldonado-Bonilla et al., 2013). This motif is present in 34.48% of the upstream sequences of the genes in the module. No enriched motifs were found in the upstream sequences of genes in module 65.

## 5 Discussion

At a rate of 378 g a.i. ha^-1^, flumioxazin is injurious to many snap bean cultivars, but not all. On average, flumioxazin decreased plant density by 84.36% and biomass per plant by 59.49%, relative to the nontreated control. The extent of injury was greater in a year when a high rainfall event occurred as seedlings were emerging. Previous studies in snap bean found crop injury from sulfentrazone, another soil applied PPO-inhibitor, varies with environmental conditions, including soil moisture (Saballos et al., 2022). In soybean, flumioxazin applied preemergence caused injury to seedlings after a rain event seven days after planting (Priess et al., 2020), but varietal differences in tolerance were observed. The increased injury we observed in 2021 compared to 2022 was likely due to the rain event that occurred eight days after planting.

Currently, flumioxazin is not registered for use in snap bean. Prior to registering an herbicide for a specialty crop, sufficient product performance and crop safety data are required (Kunkel et al., 2008). Our research demonstrates that tolerance to flumioxazin exists in snap bean; however, this tolerance is limited to certain cultivars, particularly those in the market class Romano. Although flumioxazin is unlikely to be a viable herbicide for weed control in snap bean in the immediate future, investigation of the genetic basis for this flumioxazin tolerance contributes to the broader, mechanistic understanding of plant response to xenobiotics in the environment.

Snap bean cultivar tolerance to flumioxazin is controlled by genetic factors. In this study, tolerance was expressed the most as plants germinated and emerged, and to a lesser extent, as seedlings grew. Flumioxazin tolerance measured as PDperc is more stable than tolerance measured as BPperc, with greater heritability. In addition, GWAS analysis was able to detect a genomic location that is responsible for most of the phenotypic variation for PDperc. This same region explains only a moderate amount of the variation for BPperc. This indicates that the variation of BPperc is influenced by additional genetic factors that were not identified using the strict thresholds in this analysis. Seed germination and seedling growth are two different but related physiological phenomena that are likely controlled by different sets of genes (Bentsink and Koornneef, 2008). The correlations between the traits in control conditions and under herbicide stress support the idea that the expression of the tolerance as PD and BP may be controlled by overlapping but different sets of factors. PDcontrol and PDperc had a low and negative correlation, indicating the specific factors influencing the germination of snap bean exposed to flumioxazin are different from the factors controlling germination under control conditions. In contrast, BPcontrol and BPperc were positively correlated with each other, indicating common factors influencing early biomass accumulation in both conditions. The genetics of early seedling growth are thought to be complex and involve processes related to the initial seed weight, the mobilized fraction of seed reserve, and the conversion efficiency of mobilized seed reserves to seedling tissues (Yang and Wen, 2017). Therefore, the tolerance measured as BPperc may be influenced by multiple genes with small effects that also act on the BP under control conditions, in addition to the common factor with PDperc. Flumioxazin tolerance, specially measured as PDperc, is heritable and stable. For five of the most tolerant snap bean entries, average plant density and biomass per plant reductions due to exposure to 2X the dose of flumioxazin were less than 30% and 19%, respectively. The results of this study also show that highly tolerant entries withstand flumioxazin under varied environmental conditions. The genetic variability present in the snap bean panel can be used to further understand how plants respond to flumioxazin.

In this study, tolerance to flumioxazin was largely influenced by two factors: seed weight and the presence of the favorable allele of the significant SNP. Market class “Romano” cultivars tend to have large seed size above the panel average (38.22g vs 27.30g) and are enriched for the presence of the favorable allele likely due to the prevalence of cultivar “Roma” in their pedigrees. Previous research on dry and snap beans revealed that different market classes and seed weights respond differently to various herbicides, such as flumioxazin and sulfentrazone (Urwin et al., 1996; Soltani et al., 2003, 2004, 2005, 2006; Wilson, 2005; Hekmat et al., 2007; Saballos et al., 2022). Generally, larger-seeded cultivars showed greater tolerance in these studies. This correlation between seed weight/size and herbicide tolerance has been observed in other species and herbicides as well. Larger seed size results in more robust seedlings, which have greater ability to withstand stress (Winn, 1988; Pluess et al., 2005; Ambika et al., 2014). Therefore, the link between seed size and herbicide tolerance may be due to seedling vigor. Larger seedlings are more likely to survive injury, which enables them to metabolize the herbicide and recover. However, seed size alone does not completely account for flumioxazin tolerance, as seen in the significant difference in tolerance between the populations of cultivars carrying the advantageous and disadvantageous allele of the significantly associated maker. Therefore, the genetic factors that determine flumioxazin tolerance are likely a combination of those indirectly influenced by seed size/weight and those that directly affect the herbicide’s metabolism or reduce its damage.

GWAS analysis identified a single genomic location strongly associated with tolerance, which explains a large percentage of the phenotypic variability. The high accuracy of a genomic prediction model that includes seed weight and SNP effects suggests that breeding for increase tolerance to flumioxazin is possible.

While the single genomic location associated with the tolerance could be the result of TSR mechanism, neither of the genes coding for PPO enzymes in snap bean are in the associated region. Herbicide tolerance through TSR often results in a fitness penalty for the plant (Williams et al., 1995; Vila-Aiub et al., 2009). In this study, tolerance to flumioxazin was positively correlated with seedling biomass accumulation in the nontreated control, suggesting that general mechanisms conferring seedling vigor play a role in conferring tolerance to flumioxazin. Under the hypothesis of NTSR, enhanced metabolism would degrade the herbicide before it becomes lethal to the plant. Increased hormone levels may increase seedling vigor, allowing more plants to escape herbicide toxicity. The phytotoxicity of flumioxazin is due to the creation of singlet oxygen by protoporphyrin IX in the cytoplasm. Biological processes activating antioxidant enzymes through the salicylic and jasmonic acid pathways (Ananieva et al., 2002; Kaya and Doganlar, 2016) and elevated amounts of non-enzymatic antioxidants such as tocopherols, carotenoid, glutathione, and ascorbate (Kusvuran et al., 2016) have been proposed as the underlying mechanisms of tolerance to herbicides that create oxidative stress. While we identified a miss-sense mutation in a gene located in the GWAS interval predicted to code for an oxidoreductase enzyme, no homologs of this enzyme have been directly implicated in herbicide tolerance. It is unlikely that the lack of the activity of this single enzyme is responsible for the differences in tolerance observed in this study.

Results of the network analysis support the hypothesis of NTSR mediated by general stress tolerance. We identified two main pathways associated with flumioxazin tolerance. Upregulated modules are dominated by proteins involved in oxidoreduction processes. ROS homeostasis plays a central role in abiotic stress tolerance in plants (Nadarajah, 2020; a review). Genes coding for proteins such as L-ascorbate peroxidase (gene model Phvul.002G104700), involved in the ascorbate glutathione cycle, point to the possible roles of the genes in the up-regulated modules in general stress tolerance. The module also contains a homolog of genes directly implicated in herbicide response. Phvul.010G013100 codes for a homolog of cytochrome P450 enzymes known to be responsible for the detoxification of a wide range of herbicides in maize [CYP81A9 (Nordby et al., 2008)] and the herbicide bentazon in soybean [CYP81E22 (Kato et al., 2020)]. An intriguing link between the upregulated and downregulated modules is Phvul.002G206900, coding for a receptor kinase homolog of Arabidopsis BAK1. BAK1 negatively regulates the BR- independent cell-death pathway (He et al., 2007). Double mutants of *bak1* and its paralog *bkk1* present spontaneous autoimmune response, mediated by NBS-LLR proteins (Wu et al., 2020).

Downregulated modules are dominated by NBS–LRR proteins. LRR proteins recognize specific pathogen-derived products and mediate a resistance response that includes a type of cell death known as the hypersensitive response (HR). It is tempting to derive the conclusion that the higher levels of LRR proteins result in increased cell death in sensitive plants. However, most LRR proteins are specific to their elicitor, so it would be a novel process for them to mediate a systemic cell death in response to flumioxazin. The involvement of disease resistance genes in the response to flumioxazin in snap bean needs to be elucidated.

While the network analysis offers insight into the genes and biological processes likely involved in flumioxazin tolerance, the relationship between them and the genomic interval identified by GWAS remains to be explained. The associated modules are composed of hundreds of genes in multiple chromosomes, suggesting a common regulatory element orchestrating their coordinated expression. One possibility is the presence of transcriptional factors that act in the cis-regulatory elements of the genes. To investigate this possibility, we searched for transcription factors in a set of expressed genes in or near the GWAS interval. A C2H2-like zinc finger (Phvul.002G184700) presented sequence variation predicted to create a missense mutation associated with the tolerance phenotype among 12 snap bean cultivars. C2H2 are one of the largest families of eukaryotic TF, thought to play important roles in abiotic and biotic stress resistance (Kiełbowicz-Matuk, 2012; Han et al., 2020). The upstream sequences of the set of genes composing one of the upregulated modules are enriched in a TF binding motif with high similarity to the canonical binding site for an Arabidopsis C2H2 family TF. This strengthens the hypothesis that the putative C2H2 family transcription factor located in the GWAS interval is controlling the expression of the genes in the tolerance associate module. Follow up studies will be needed to elucidate the role of Phvul.002G184700 in the regulation of genes associated with tolerance to flumioxazin.

In summary, our results suggest that tolerance to flumioxazin in snap bean is mediated by upregulation of oxidoreductase activities and downregulation of apoptotic pathway, likely controlled by a master element located in chromosome 2. The existence of a single element able to regulate the expression of a large number of genes involved in stress tolerance is of high interest for basic science and applied breeding. Stress-resistant traits are often complex with the involvement of multiple genes. The existence of cultivars with high breeding value for the tolerance trait in several market classes, combined with the relative easiness of introgressing a single region through marker assisted selection or manipulation through gene editing, can facilitate the creation of flumioxazin tolerant cultivars. Conceivably, excessive reliance on flumioxazin or related herbicides for weed control could select for phenotypes of weed species that are enriched in tolerance mechanisms identified in this research.

## Data availability statement

The data presented in the study are deposited in the Gene Expression Omnibus genomics data repository supported by the National Center for Biotechnology Information, GEO accession numbers: GSM8342381 - GSM8342426 and GSE270416.

## Author contributions

AS: Conceptualization, Data curation, Formal analysis, Methodology, Writing – original draft, Writing – review & editing. MB: Formal analysis, Visualization, Writing – review & editing. PT: Methodology, Writing – review & editing. MW: Conceptualization, Funding acquisition, Supervision, Writing – review & editing.
