# Supplementary material for: Mapping of flumioxazin tolerance in a snap bean diversity panel leads to the discovery of a master genomic region controlling multiple stress resistance genes
Source: Front Plant Sci. 2024 Jul 2;15:1404889. doi: 10.3389/fpls.2024.1404889 (PMC11250381; doi:10.3389/fpls.2024.1404889)

Supplemental figure 1. Environmental conditions through three weeks after planting for field experiments conducted in 2021 and 2022 near Urbana, IL. A) cumulative growing degree days, and B) cumulative water supply (rainfall + irrigation).

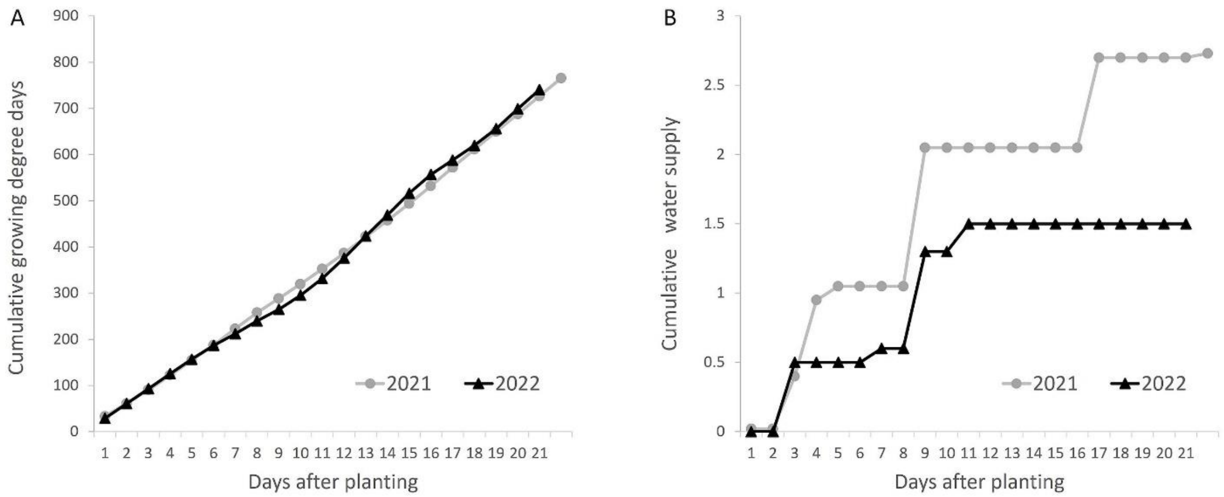

Supplemental figure 2. Histograms of original data. Plant density of flumioxazin treated plots as a percentage of the control plots (PDperc), for A) both years, C) 2021, and E) 2022. Biomass per plant of flumioxazin treated plots as a percentage of the control plots (BPperc), for B) both years, D) 2021, and F) 2022, respectively. S-W: p-value of the Shapiro-Wilk test statistic under the hypothesis of normality of the traits' distribution.

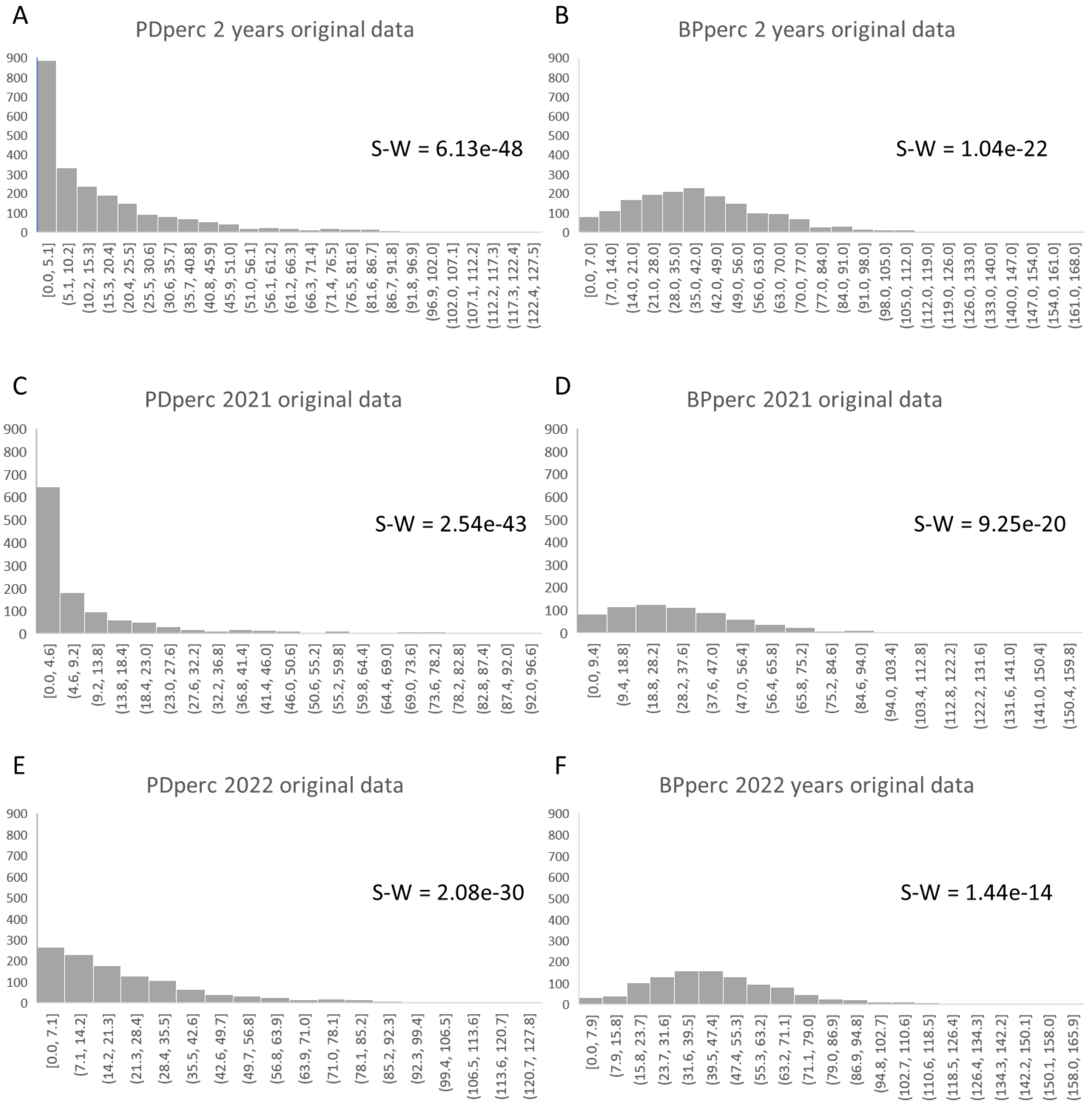

Supplemental figure 3. Histograms of Box-Cox transformed data. Plant density of flumioxazin treated plots as a percentage of the control plots (PDperc), for A) both years, C) 2021, and E) 2022. Biomass per plant of flumioxazin treated plots as a percentage of the control plots (BPperc), for B) both years, D) 2021, and F) 2022, respectively. S-W: p-value of the Shapiro-Wilk test statistic under the hypothesis of normality of the traits' distribution.

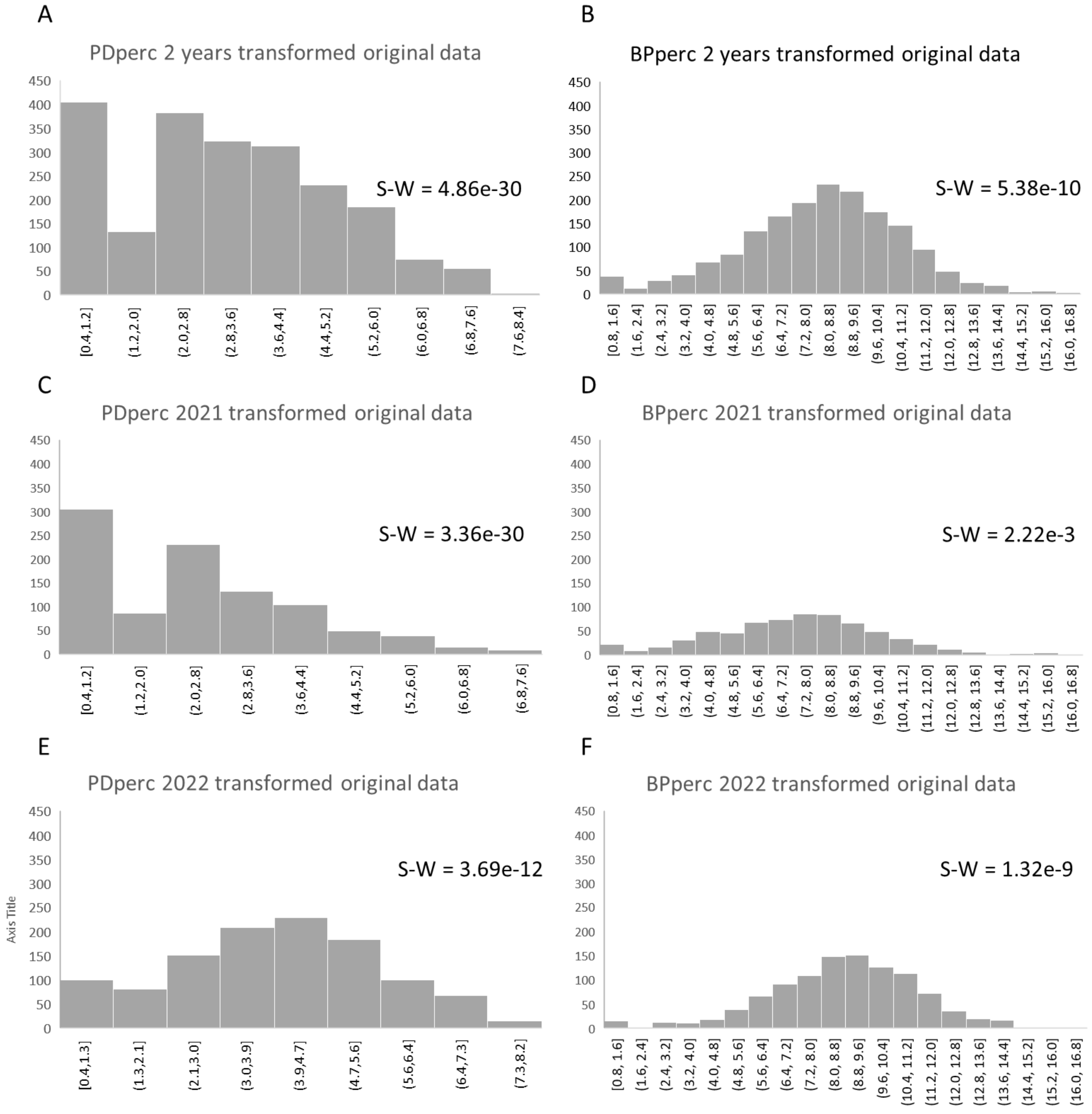

Suplemental figure 4. Linkage Disequilibrium (LD) decay in the SnAP. LD is measured as r^2 for pairwise markers. The average r^2 of 10 Kbp bins was plotted against the distance between markers.

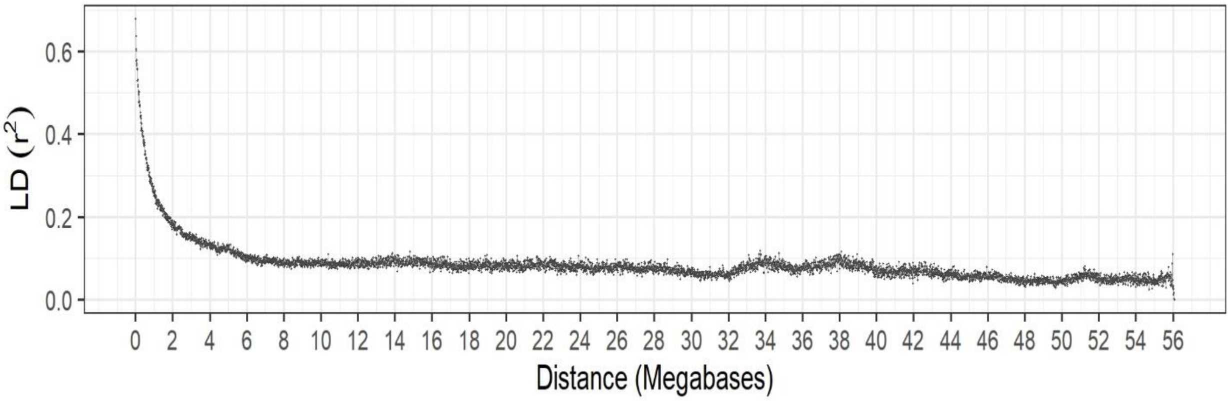

Supplemental figure 5. Enriched gene ontology areas in the set of genes upregulated in tolerant cultivars. A- Biological processes. B- Cellular component. C-Molecular function. The size of the block corresponds to the significance of the enrichment. Blocks of the same color belong to a common parent term. Parent terms are despicted in blue font, child terms are in black fond.

A

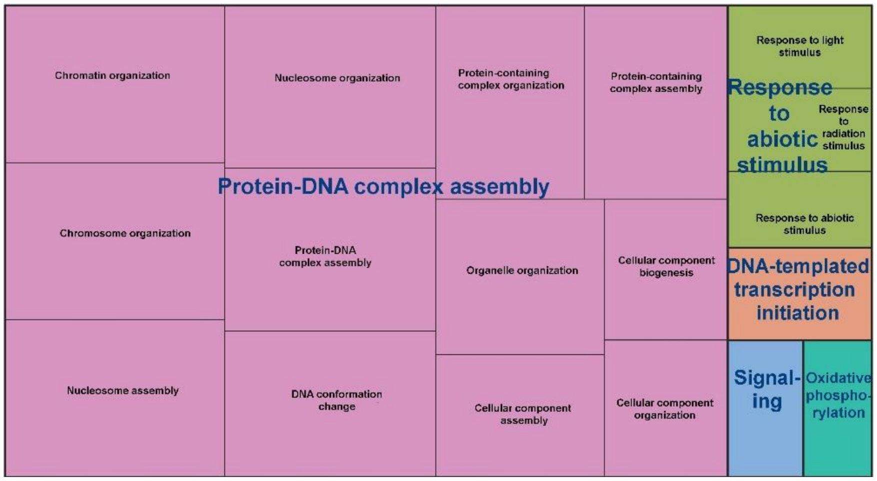

B

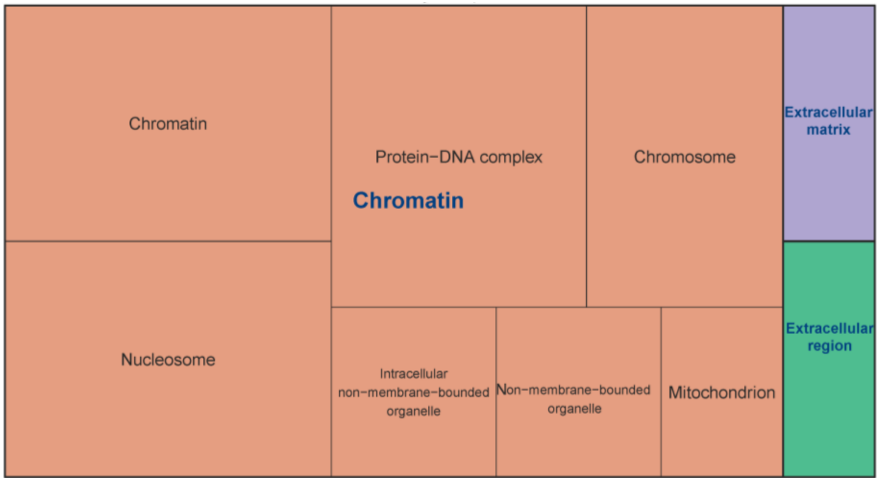

C

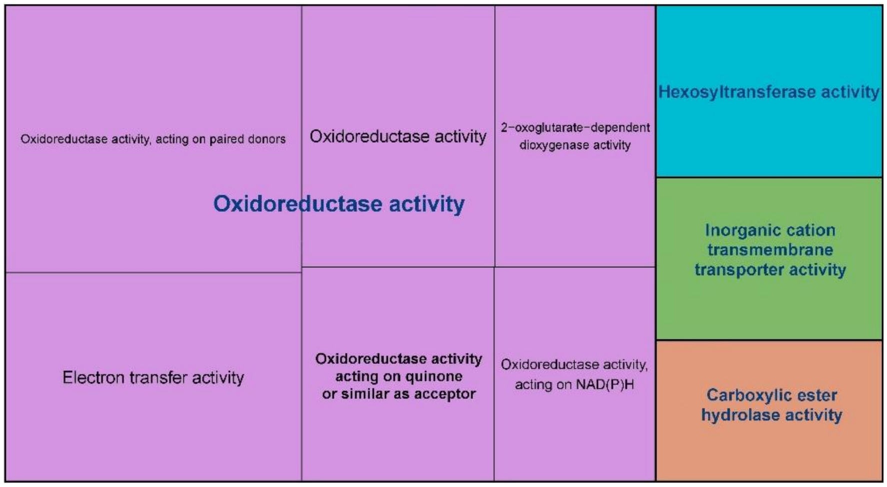

Supplemental figure 6. Enriched gene ontology areas in the set of genes downregulated in tolerant cultivars. A- Biological processes. B- Cellular component. C-Molecular function. The size of the block corresponds to the significance of the enrichment. Blocks of the same color belong to a common parent term. Parent terms are despicted in blue font, child terms are in black fond.

A

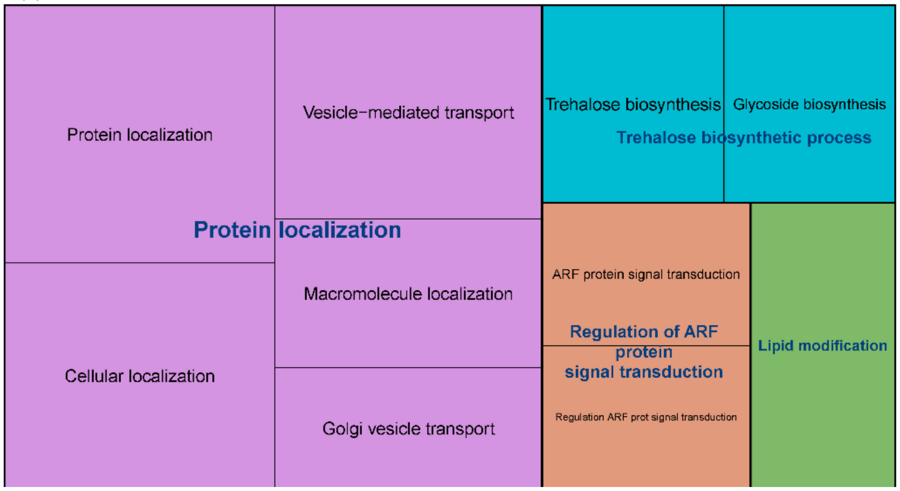

B

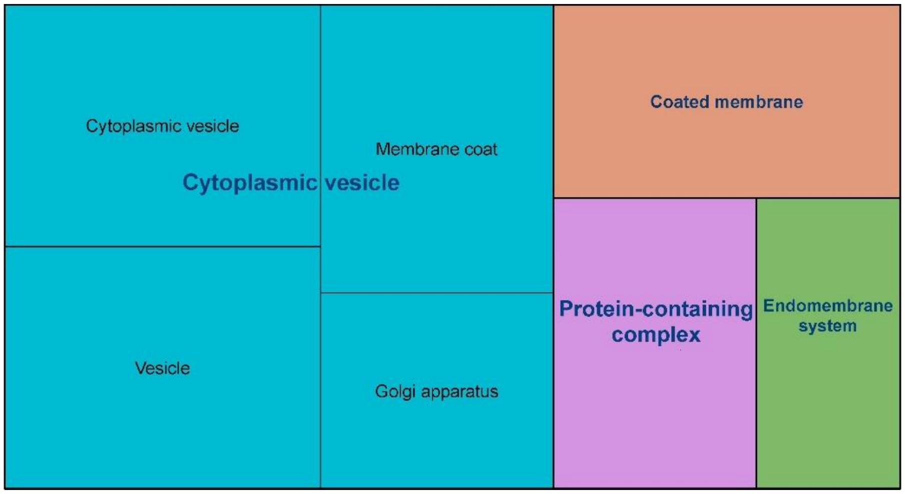

C

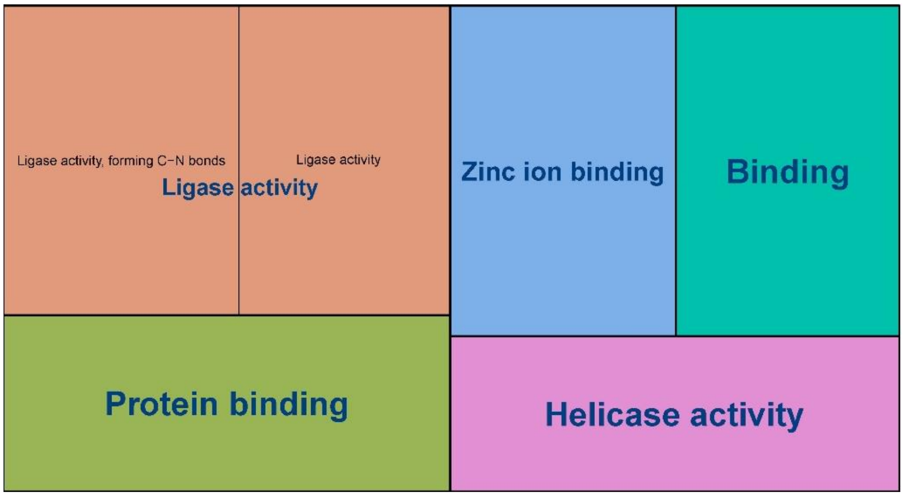

Supplemental figure 7. Results of network analysis contrasting co-expressed gene modules with differential expression between tolerant and sensitive snap bean cultivars. A) Boxplot graphs of the eigengene values each between tolerant and sensitive samples. B) Heatmaps summarizing the normalized gene expression of the genes in each module between the tolerant and sensitive samples. The bar plot at the bottom of the heatmap represents the summarized gene expression of all the genes in the module for an individual sample.

Module 16

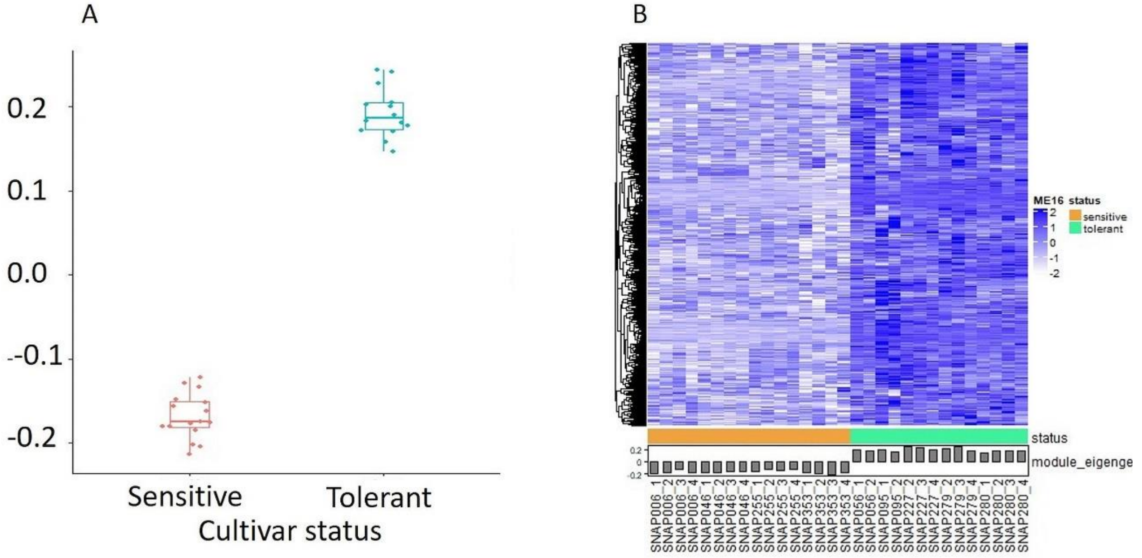

Module 59

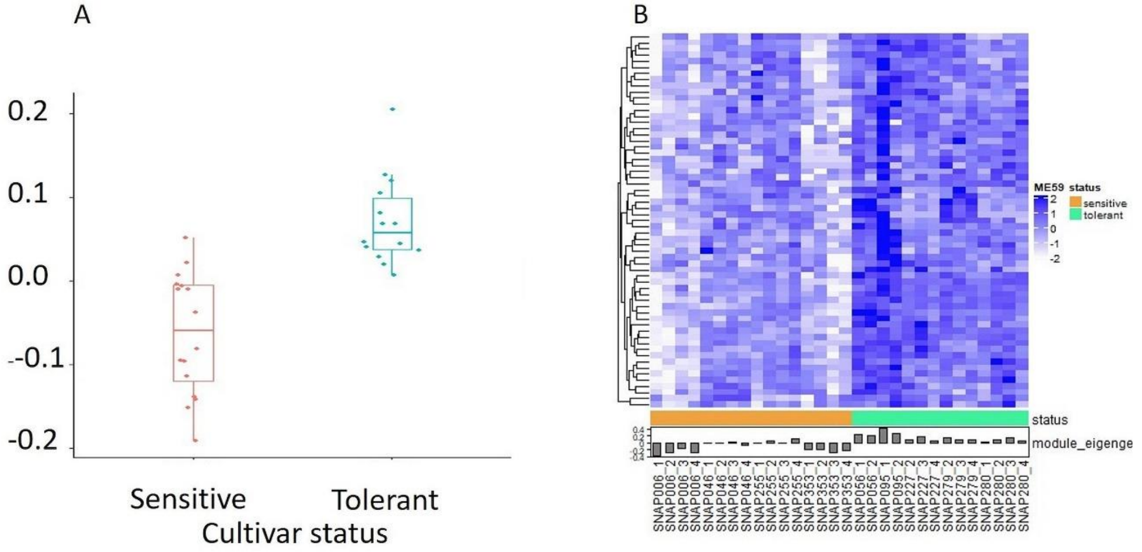

Supplemental figure 7. Continuation.

Module 18

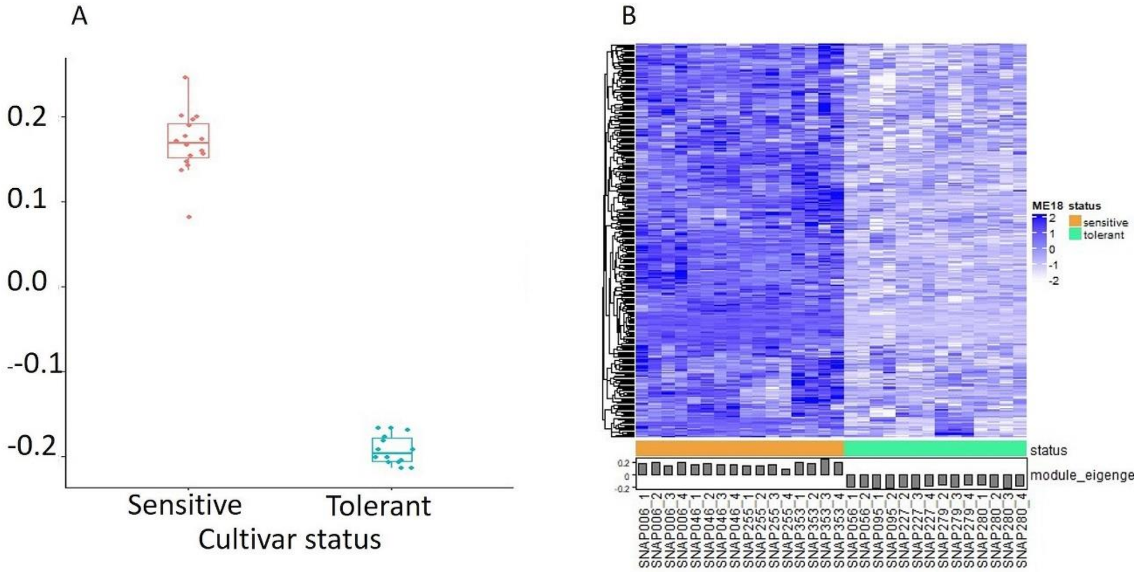

Module 65

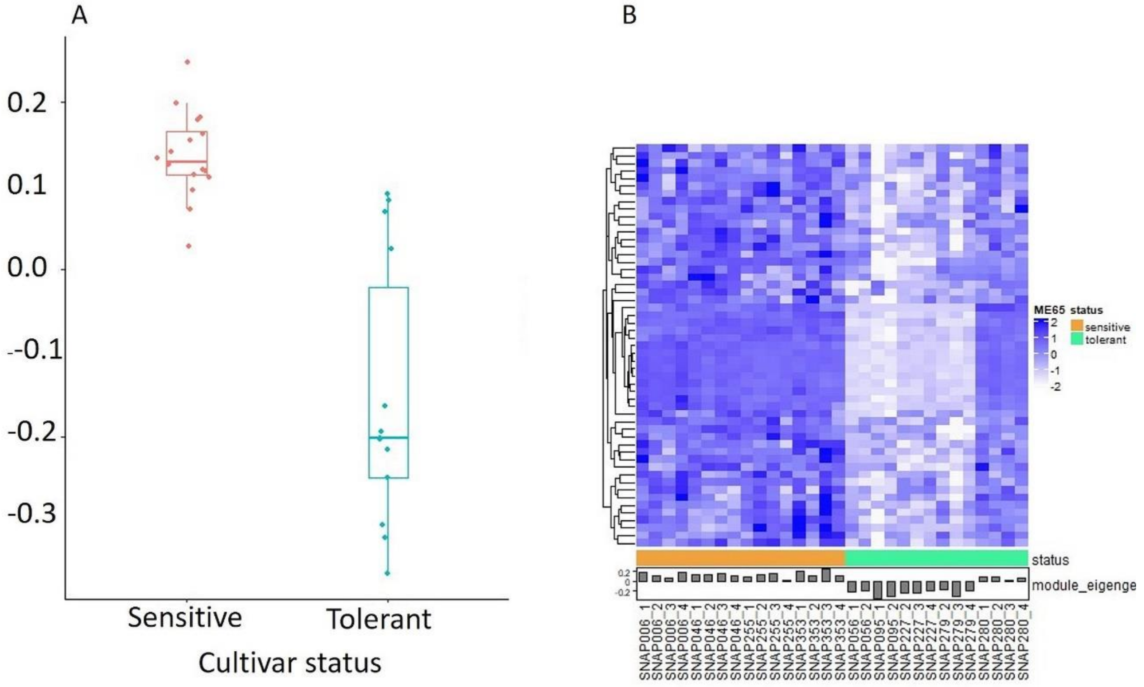

Supplemental figure 8. Pearson's correlation coefficients between the cultivars' average eigengene value of significant gene modules and the cultivars' average PDperc value. Samples from sensitive cultivars (n=4): orange circles. Samples from tolerant cultivars (n=5): light blue circles. Average eigengene value of each cultivar: x symbol, colored orange or blue for sensitive and tolerant cultivars, respectively. r_S: correlation coefficient between the variables in the sensitive cultivars group. r_T: correlation coefficient between the variables in the tolerant cultivars group. $r_{Overall}$: correlation coefficient for all cultivars (n=9). An asterisk after the *r* value indicates significance at $\alpha \leq 0.05$.

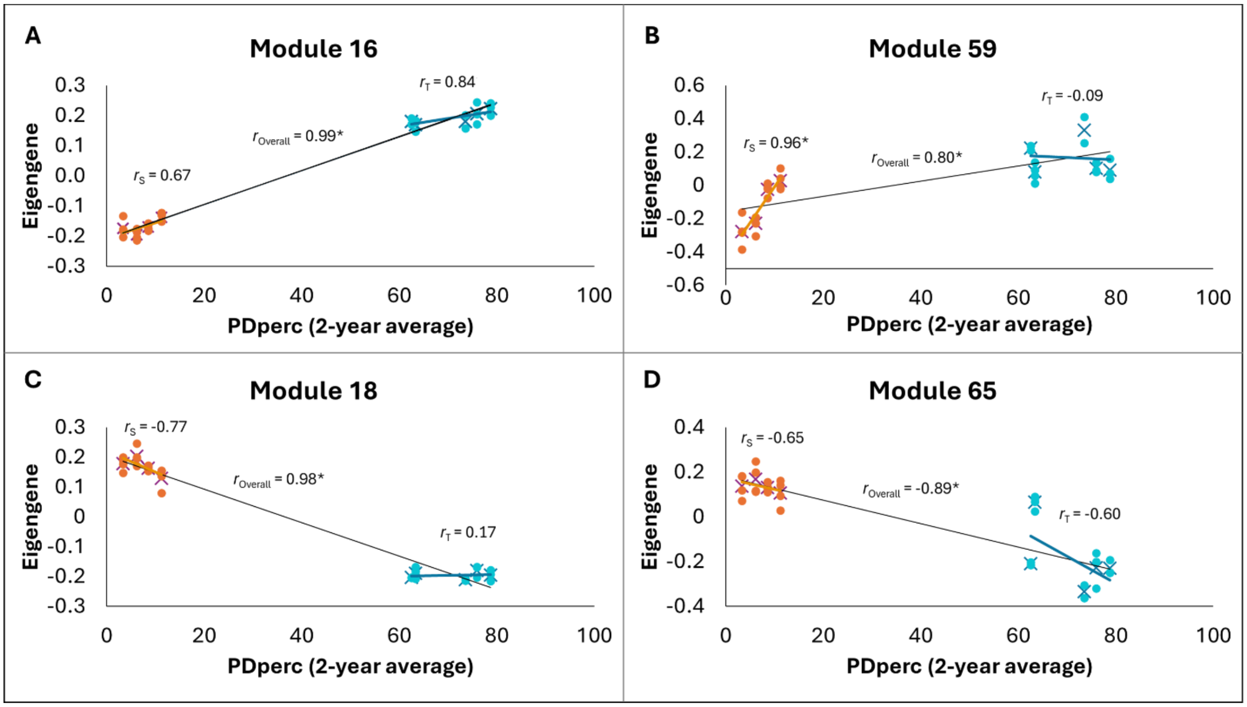

Supplement: Supplementary file 1 [file Presentation_1.pdf]
