# Supplementary material for: Mapping of flumioxazin tolerance in a snap bean diversity panel leads to the discovery of a master genomic region controlling multiple stress resistance genes
Source: Front Plant Sci. 2024 Jul 2;15:1404889. doi: 10.3389/fpls.2024.1404889 (PMC11250381; doi:10.3389/fpls.2024.1404889)
Supplement: Supplementary file 2 [file Table_1.pdf]

Supplemental table 1. Snap bean cultivars included in the RNA sequencing experiment to determine intrinsic differences in gene expression between tolerant and sensitive cultivars during seed germination.

Cultivar	SnAPID	SeedWt	PDperc	BPperc	S02_34499796	Reaction
Amigo	SnAP006	43.40	3.33	29.65	AA	Sensitive
Top Crop	SnAP353	44.29	6.15	14.86	AA	Sensitive
Bounty	SnAP046	43.49	8.52	23.37	AA	Sensitive
Pirate	SnAP255	46.07	11.18	24.15	AA	Sensitive
Kentucky Wonder Pole	SnAP190	47.46	11.45	46.78	AA	Sensitive
Golden Gate Wax	SnAP153	50.33	15.07	42.00	AA	Sensitive
Moncayo	SnAP227	42.44	78.72	50.70	GG	Tolerant
Roma II	SnAP279	47.29	75.90	68.20	GG	Tolerant
DMC 06-01	SnAP095	45.35	73.55	63.52	GG	Tolerant
Bountiful	SnAP045	45.81	70.82	52.97	GG	Tolerant
Romano 118	SnAP280	47.69	63.33	59.90	GG	Tolerant
Bush Romano 350	SnAP056	45.81	62.50	98.10	GG	Tolerant

Supplemental table 2. Alignment rate for the samples included in the RNS sequencing experiment.

Sample	Sequenced Reads	Filtered Reads	Alignment Rate
SNAP006_1	66,494,258	66296834	96.20%
SNAP006_2	58,038,860	57816528	95.84%
SNAP006_3	73,075,526	72798360	95.29%
SNAP006_4	53,616,478	53423604	95.57%
SNAP045_1	61,784,072	61743174	95.91%
SNAP045_2	68,538,656	68449690	95.80%
SNAP045_3	67,488,492	67411092	96.10%
SNAP045_4	68,866,180	68817352	96.17%
SNAP046_1	57,960,750	57771466	95.99%
SNAP046_2	73,086,094	72897678	95.76%
SNAP046_3	56,291,728	56194982	96.26%
SNAP046_4	66,315,994	66198398	95.02%
SNAP056_1	82,062,002	81779924	96.12%
SNAP056_2	54,120,824	53892642	96.06%
SNAP056_3	51,489,774	51124224	95.58%
SNAP056_4	66,615,614	66076526	93.97%
SNAP095_1	68,031,244	67948848	95.66%
SNAP095_2	72,082,014	71999892	95.98%
SNAP095_3	66,582,814	66374371	95.34%
SNAP095_4	59,264,254	59217332	95.44%
SNAP153_1	55,503,838	55451802	96.42%
SNAP153_2	55,614,066	55536400	96.22%
SNAP153_3	66,344,888	66244900	96.15%
SNAP190_1	68,912,546	68869330	89.24%
SNAP190_2	87,303,282	87263296	96.86%
SNAP190_3	74,292,766	74226424	92.33%
SNAP190_4	56,566,502	56538670	96.45%
SNAP227_2	55,399,204	55237268	95.80%
SNAP227_3	55,384,020	55238604	96.21%
SNAP227_4	58,763,152	58635214	96.36%
SNAP255_1	57,330,404	57142072	95.14%
SNAP255_2	57,364,214	57144974	95.59%
SNAP255_3	66,275,356	66061332	95.67%
SNAP255_4	77,936,532	77758304	96.28%
SNAP279_1	56,509,572	55463098	93.58%
SNAP279_2	65,150,904	64953484	95.97%
SNAP279_3	56,326,606	55851940	95.05%
SNAP279_4	60,318,348	60020270	95.31%
SNAP280_1	51,599,754	51415132	95.72%
SNAP280_2	70,966,258	70710018	96.09%
SNAP280_3	58,551,592	58289282	95.90%
SNAP280_4	58,767,634	58610972	96.03%

SNAP353_1	70,257,136	70201096	96.54%
SNAP353_2	66,776,382	66713162	96.18%
SNAP353_3	85,315,682	85209896	95.69%
SNAP353_4	60,891,472	60796152	95.86%
